# Breast cancer-derived exosomes transmit lncRNA SNHG16 to induce CD73+γδ1 Treg cells

**DOI:** 10.1038/s41392-020-0129-7

**Published:** 2020-04-29

**Authors:** Chao Ni, Qing-Qing Fang, Wu-Zhen Chen, Jin-Xing Jiang, Zhou Jiang, Jun Ye, Ting Zhang, Liu Yang, Fan-Bo Meng, Wen-Jie Xia, Miaochun Zhong, Jian Huang

**Affiliations:** 1 Department of Breast Surgery, Second Affiliated Hospital, Zhejiang University, Hangzhou, Zhejiang 310009 China; 2 Key Laboratory of Tumour Microenvironment and Immune Therapy of Zhejiang Province, Second Affiliated Hospital, Zhejiang University, Hangzhou, Zhejiang 310009 China; 3 Key Laboratory of Tumour Molecular Diagnosis and Individualized Medicine of Zhejiang Province, Zhejiang Provincial People’s Hospital, People’s Hospital of Hangzhou Medical College, Hangzhou, Zhejiang 310014 China; 4 Department of Thyroid and Breast Surgery, Affiliated Hospital of Shaoxing University, Shaoxing, Zhejiang 312099 China; 5 Department of Thyroid and Breast Surgery, Zhejiang Provincial People’s Hospital, People’s Hospital of Hangzhou Medical College, Hangzhou, Zhejiang 310014 China

**Keywords:** Breast cancer, Tumour immunology

## Abstract

γδT cells have been reported to exert immunosuppressive functions in multiple solid malignant diseases, but their immunosuppressive functional subpopulation in breast cancer (BC) is still undetermined. Here, we collected 40 paired BC and normal tissue samples from Chinese patients for analysis. First, we showed that γδT1 cells comprise the majority of CD3+ T cells in BC; next, we found that CD73+γδT1 cells were the predominant regulatory T-cell (Treg) population in BC, and that their prevalence in peripheral blood was also related to tumour burden. In addition, CD73+γδT1 cells exert an immunosuppressive effect via adenosine generation. We also found that BC could modulate CD73 expression on γδT cells in a non-contact manner. The microarray analysis and functional experiments indicated that breast tumour cell-derived exosomes (TDEs) could transmit lncRNA SNHG16, which upregulates CD73 expression, to Vδ1 T cells. Regarding the mechanism, SNHG16 served as a ceRNA by sponging miR-16–5p, which led to the derepression of its target gene SMAD5 and resulted in potentiation of the TGF-β1/SMAD5 pathway to upregulate CD73 expression in Vδ1 T cells. Our results showed that the BC-derived exosomal SNHG16/miR-16–5p/SMAD5-regulatory axis potentiates TGF-β1/SMAD5 pathway activation, thus inducing CD73 expression in Vδ1 T cells. Our results first identify the significance of CD73+Vδ1 Tregs in BC, and therapy targeting this subpopulation or blocking TDEs might have potential for BC treatment in the future.

## Introduction

Although breast cancer (BC) is recognised as a “cold” tumour compared with other solid malignant diseases with strong immunogenicity, such as melanoma, lung cancer and colorectal cancer, scientists still focus on immune therapy and the immune microenvironment of BC, and the immune ecosystem within BC has been better elucidated recently with the advent of single-cell RNA-seq analysis^1^ and mass cytometry technology.^2^ Researchers have found that some immune subpopulations are highly associated with prognosis and have the potential to be therapeutic targets.

Among these subpopulations, γδT cells have been reported as a major component of tumour-infiltrating lymphocytes (TILs) in BC and are strongly correlated with unfavourable pathological characteristics and poor prognosis.^3^ Wang and Peng et al.^3,4^ first determined that γδT1 cells but not γδT2 cells were recruited from peripheral blood via the CXCR3/IP10 axis, were the dominant cell type in the CD3+ T-cell subsets in breast TILs, and functioned as regulatory immune cells via a unique TLR8 signalling pathway. In addition, we first found the protumour function of γδT17 cells in a solid tumour (colorectal cancer) in humans,^5^ and in recent years, several reports have attributed protumoural functions to γδT17 cells in BC using various inducible animal models.^6^ Although solid evidence has been presented that infiltrating γδT cells play a negative role in BC, and considering the crucial role of γδT cells in the innate and adaptive immune systems, it is rational to determine a specific marker for the identification of regulatory infiltrating γδT cells in BC.

Numerous studies have indicated that crosstalk between infiltrating immune cells, such as macrophages, B cells and cancer cells, orchestrates the BC microenvironment to facilitate tumour progression, chemoresistance and metastasis.^7,8^ In recent years, endosome-derived multivesicular bodies (MVBs) known as exosomes (usually with a diameter of 50–100 nm) have generated great interest in cancer research. At first, exosomes were thought to be functioned only in the removal of unnecessary molecules within cells, but an abundance of fascinating studies demonstrated that they could transfer their content (e.g., protein and non-coding RNA) from one cell to another and function as a regulator.^9^

In this report, we revealed that CD73+γδT1 cells were the dominant regulatory T cells (Tregs) in BC and were induced by BC cells. Furthermore, we found that BC cells promoted SMAD5 expression in γδT1 cells via transfer of exosomal long non-coding RNA (lncRNA) SNHG16, which functioned as a ceRNA by sponging miR-16–5p and therefore potentiated the TGF-β1/SMAD5 pathway to upregulate CD73 levels. Our results suggest that targeting CD73+γδT1 cells or blocking tumour cell-derived exosomes (TDEs) could be a strategy for BC treatment in the future.

## Materials and methods

### Cell lines and clinical specimens

The human BC cell lines MCF-10A, MCF-7, T-47D and MDA-MB-231 and the HEK293T cell line were purchased from the Cell Bank of the Chinese Academy of Sciences (Shanghai, China). MCF-10A cells were cultured with an MEGM kit (Lonza/Clonetics, CC-3150); all other cells were maintained in a humidified CO2 incubator at 37 °C in DMEM supplemented with 10% foetal bovine serum (FBS) and 1% penicillin–streptomycin.

All human BC tissues and paired normal breast tissue samples were obtained from 40 patients with BC who underwent modified mastectomy or breast-conserving surgery between 2014 and 2016 at Zhejiang Provincial People’s Hospital. Five to 10 ml of peripheral blood from BC patients who provided formal consent was collected before and after surgery. Peripheral blood samples were also obtained at Zhejiang Blood Centre from healthy donors, all of whom were negative for antibodies against hepatitis C virus, hepatitis B virus, HIV and syphilis. The collection of all study-related clinical samples was approved by the ethics committees of Zhejiang Provincial People’s Hospital. Informed consent was obtained from all enrolled subjects, and the study was performed in full compliance with the principles of the Helsinki Declaration.

### Cell isolation and culture

For isolating γδT cells in breast tissue, fresh specimens were thoroughly washed twice in PBS containing 10× penicillin and streptomycin (Invitrogen), cut into small pieces (1 mm^3^) with sterile scalpel blades and digested in RPMI 1640 (Invitrogen) medium supplemented with 2% FBS, 1 mg/mL type IV collagenase and 1 mg/mL hyaluronidase (Sigma) for 4 h at 37 °C to obtain a single-cell suspension. Tissue debris was removed by passing the suspension through a 100-μm filter. CD4+ Tregs (CD3+CD4+CD25+), Vδ1 T cells (CD3+Vδ1+), Vδ2 T cells (CD3+Vδ2+) and CD73+Vδ1 T cells (CD3+CD73+Vδ1+) were sorted by a FACS Aria II cell sorter (BD Biosciences). For isolating CD3+, Vδ1 T and Vδ2 T cells in the peripheral blood from healthy donors, peripheral blood mononuclear cells (PBMCs) were obtained with Ficoll (1.077 g/ml) density-gradient centrifugation at 400 × *g* for 15 min. Then, the cell pellet was resuspended in PBS, labelled with corresponding antibodies and processed with a FACS Aria II cell sorter (BD Biosciences) to separate the different populations. The purity of all the sorted cells was >90%.

### Flow cytometry

For extracellular surface marker staining, single-cell suspensions were obtained from peripheral blood or breast tissues. Cells were suspended in cell staining buffer (BioLegend) and incubated with various combinations of fluorochrome-coupled antibodies (Supplementary Table S1). For intracellular staining, lymphocytes were activated by Leukocyte Activation Cocktail (BD Pharmingen) for 6 h following the manufacturer’s protocol. Cells were collected on a FACSCanto II system, and the data were analysed with FlowJo software (TreeStar). Due to the limited number of Vδ1 T cells, we collected an equal number of 5000 live cells from the intracellular staining and co-culture experiments for FACS analysis.

### Cell proliferation, in vitro cytotoxicity assay and blocking assay

For proliferation assays, CD3+ T cells were isolated and labelled with CFSE and then co-cultured with specific cells (CD73+Vδ1 T and CD73-Vδ1 T cells) in the RPMI 1640 medium supplemented with IL-2 (40 U/ml, Peprotech), anti-CD3 antibody (10 μg/ml, clone UCHT1, BioLegend) and anti-CD28 antibody (10 μg/ml, clone CD28.2, BioLegend). CD3+ T cells were harvested, and CFSE^low^ CD3+ T cells were detected by flow cytometry at day 6.

The cytotoxicity experiment of γδT cells against BC cells was performed with the CellTrace Far Red DDAO-SE kit (Invitrogen) according to the manufacturer’s protocol. γδT cells and DDAO-SE-labelled BC cells were co-incubated at different effector:target (E:T) ratios (1:1, 5:1, 10:1) for 4 h. Then, PI (1 mg/mL, BD Biosciences) was added to the medium for another 15 min, and DDAO-SE+ PI+ cells were analysed by flow cytometry.

To investigate the effects of adenosine, IL-10 and TGF-β on the immunosuppressive effect of CD73+Vδ1 T cells, CD3+ T cells were either the pre-incubated with A2A (0.1 mM, SCH58261, MCE) and A2B (PSB603, CAS No. 1092351–10–4, 0.05 mM) receptor antagonists or were treated with neutralisation antibodies against IL-10 (1 μg/ml, cone JES3–9D7, BioLegend) and TGF-β1 (1 μg/ml, clone 9016.2, Genetex) in the medium. Then, the proliferation of CFSE-labelled CD3+ T cells was evaluated by flow cytometry on day 6 after treatment. To explore the effect of TGF-β1 and BMP4 on CD73 expression in Vδ1 cells, recombinant TGF-β1 (10 ng/mL) or BMP4 (10 ng/mL) (R&D Systems) was added to the medium, and the cells were pretreated with or without SB-431542 (Sigma-Aldrich; 10 μm, 1 h) or dorsomorphin (Sigma-Aldrich; 10 μm, 1 h).

### Extracellular adenosine detection

The adenosine concentration in the supernatant of homogenates from tumour and paired normal tissues and in the media from cultured cells, both of which were diluted 100×, were assessed with an adenosine assay kit (Abcam). The fluorescence intensity was measured at Ex/Em 535/587 using a fluorescence spectrophotometer (Agilent Technologies, CA, USA).

### ELISA

To compare the immunosuppressive functions of CD73+Vδ1 T, CD73-Vδ1 T and CD4+CD25+ T cells, the corresponding cells were sorted from BC specimens and then co-cultured with allogeneic CD4+ or CD8+ T cells from peripheral blood in the presence of IL-2 (40 U/ml, Peprotech), anti-CD3 antibody (10 μg/ml, clone UCHT1, BioLegend) and anti-CD28 antibody (10 μg/ml, clone CD28.2, BioLegend). The IFN-γ (BioLegend), Perforin (Abcam) and Granzyme B (BioLegend) levels were detected with corresponding ELISA kits.

### Exosome isolation and transfer assay

Cells in the logarithmic growth phase were collected and seeded into 10-cm culture dishes. When the cells were ~70% confluent, the cell culture medium was replaced with exosome-free serum. After another 48 h of culture, the cell culture supernatant was collected and subjected to gradient centrifugation (300 × *g*, 10 min; 2000 × *g*, 20 min; 10,000 × *g*, 30 min), after which the supernatant underwent ultracentrifugation (100,000 × *g*, 70 min). Precooled PBS was used to resuspend the resulting pellet, and the suspensions were centrifuged again by ultracentrifugation (100,000 × *g*, 70 min). The supernatant was resuspended in 50 µl of precooled PBS and stored at −80 °C. All of the above steps were performed at 4 °C.

Exosomes derived from breast cancer cells (BCCs) were isolated by ultracentrifugation, resuspended in PBS supplemented with PKH26 (2 μm, 37 °C, 10 min) in the dark, co-cultured with Vδ1 T cells for 24 h and analysed by flow cytometry thereafter.

### Microarray analysis

The differential expression of exosomal lncRNAs between a normal breast cell line (MCF-10A) and BCC lines (MCF-7 and MDA-MB-231) was analysed by LC Sciences (LC Biotech Human lncRNA Microarray 4 × 180 K). Differentially expressed lncRNAs were identified using stringent filtering criteria (fold change ≥ 4, *p* < 0.05). Heatmaps were generated with “Multiple Array Viewer” (version 4.9.0).

### CIBERSORT and bioinformatic analysis

To evaluate the prognostic value of tumour-infiltrating γδT cells in breast cancer, we conducted a systematic search in GEO data sets (https://www.ncbi.nlm.nih.gov/gds) to identify breast cancer gene expression data sets with available clinicopathological information. After screening, data sets based on the GPL570 platform ([HG-U133_Plus_2] Affymetrix Human Genome U133 Plus 2.0 Array) were included for further analysis. The CIBERSORT-LM7 deconvolution algorithm was applied to estimate the abundance of γδT cells based on the mRNA expression profile data.^10^ All statistical analyses were conducted using R studio software (version 1.1.414; http://www.rstudio.com/products/rstudio). A Cox proportional hazards regression model was used to calculate hazard ratios (HRs) and 95% confidence intervals (95% CIs). The Kaplan–Meier method with log-rank test was used to compare survival curves between groups, and multivariate Cox regression analysis was also performed with overall survival (OS) and disease-free survival (DFS) as the outcome variables.

Based on the same data sets, breast cancer patients were divided into three groups based on the relative abundance of γδT cells. We compared the differences in age, T stage and N stage among these three groups. The measurement data are expressed as the means ± standard deviation, and the count data are expressed as frequency (percent). The difference analysis was performed with the chi-square test, and *p* < 0.05 was defined as a significant difference.

### Immunoblotting

Protein samples were boiled in SDS/β-mercaptoethanol sample buffer, and 20 μg of protein from each sample was loaded on a gel. The antibodies used for western blotting were rabbit anti-CD9 (Abcam), rabbit anti-CD63 (Abcam) and rabbit anti-SMAD5 (Proteintech). Anti-GAPDH antibody (Proteintech) was used to detect GAPDH, which served as a loading control.

### RNA extraction and RT-qPCR

The total RNA was extracted from tissues and cultured cells with TRIzol (Invitrogen, Carlsbad, CA, USA) in accordance with the manufacturer’s instructions. Approximately 1 μg of the total RNA was reverse transcribed to cDNA using a reverse transcriptase cDNA synthesis kit (Toyobo, Osaka, Japan), and qPCR was performed using a SYBR Green PCR kit (Roche, Basel, Switzerland). Comparative quantification was assessed using the 2^−ΔCt^ method with GAPDH as the endogenous control. All primers used are listed in Supplementary Table S2.

### Plasmid construction and siRNA silencing

A full-length 2435 bp sequence was cloned into the pCR3.1 vector to construct the SNHG16 overexpression vector. The sequences of SNHG16-specific siRNAs were as follows: SNHG16-homo-349, 5′­GCCUCUGCUGCUAAUUGUUTT-­3′; SNHG16-homo-867, 5′-­CCAAGGAGGGACUGUUUAATT­-3′ and SNHG16-homo-2004, 5′-CCCAGUGUUGACUCACCAATT­-3′. The miR-16–5p mimics, inhibitor and negative controls were purchased from GenePharma (Supplementary Table S3).

To manipulate the expression of miR-16–5p in Vδ1 T cells, Vδ1 T cells were sorted from peripheral blood and seeded into six-well plates at 1 × 10^6^ cells/well with 1 ml of medium supplemented with 10% FBS, 10 μg/ml CD3, 10 μg/ml CD28 and 40 U/mL IL-2. Then, transfection reagent (INVI DNA RNA Transfection Reagent, Invigentech, USA) and miR-16–5p mimics/NC/inhibitor/inhibitor NC (20 μm) were incubated with the cells for 48 h, after which the cells were collected for further experiments.

### Dual-luciferase reporter assays

HEK293T cells were seeded in 96-well plates at a density of 1 × 10^4^ cells per well for 24 h before transfection. The cells were co-transfected with 25 μl of the miRNA mimics (20 μm, Supplementary Table S3), 0.1 μg of the SMAD5 or SNHG16 promoter-containing luciferase reporter plasmid and 0.1 μg of pRL-TK plasmid per well with Lipofectamine 3000 (Invitrogen, Carlsbad, CA, USA). PRL-TK was used as the internal control. Approximately 48 h after transfection, the cells were subjected to luciferase activity analysis using a Dual-Luciferase Reporter Assay System (Promega) following the manufacturer’s instructions.

### RNA-binding protein immunoprecipitation assay

RNA immunoprecipitation (RIP) assays were performed with the Magna RIP RNA-Binding Protein Immunoprecipitation kit (Millipore, USA) according to the manufacturer’s protocol. Briefly, Vδ1 T cells were lysed in lysis buffer with protease inhibitor cocktail and RNase inhibitor, and the lysates were immunoprecipitated with anti-Ago2 and IgG antibodies. Finally, the amount of retrieved RNAs was quantified by qPCR, and the relative primers used are listed in Supplementary Table S2.

### RNA in situ hybridisation

For fluorescence in situ hybridisation (FISH) assays, cells were first grown on glass cover slips in 24-well plates for 24 h. Then, we used the reagents and methods provided by the RNA FISH probe kit (GenePharma, Shanghai, China). After immobilisation and permeabilization, the cells were hybridised with 20 μm Cy3-labelled SNHG16 (probe sequence 5′-GGCCGACCTCGAAAAGAACGCTCAACACCGACTGGAGTGGCATCATCACTAAAGGCCTGA-3′) or 20 μm FITC-labelled miR-16–5p (probe sequence 5′-CGCCAATATTTACGTGCTGCTA-3′). After nuclear staining with DAPI, images were captured by confocal microscopy (Leica) at 630× magnification.

### Transmission electron microscopy

Exosomes were prepared by the abovementioned ultra-high-speed centrifugation method. The pellet was resuspended in 100 µl of PBS and fixed with an equal volume of 4% polyformaldehyde. The exosome suspension was then added to the copper net. After 5 min, the liquid was absorbed and then stained with uranium acetate for 5 min. The copper mesh was rinsed several times in ultrapure water with ophthalmic tweezers, and the liquid was allowed to dry. The copper mesh was observed under a transmission electron microscope (TECNA1–10, Philips, Netherlands).

### Statistical analysis

All data are presented as the means ± SD of at least three independent experiments, and were statistically analysed with GraphPad Prism 5 software (San Diego, CA, USA). Differences between two groups were compared using paired *t* tests (paired data) or unpaired *t* tests (unpaired data). One-way analysis of variance followed by Tukey’s post hoc test was performed for comparisons among multiple groups. The correlation between adenosine levels and CD73+Vδ1 T cells was assessed using Spearman’s correlation coefficient. A two-tailed test was performed, and a *p*-value < 0.05 was considered statistically significant.

## Results

### γδ1 T cells are the key tumour-infiltrating lymphocytes in breast cancer

**Fig. 1:**
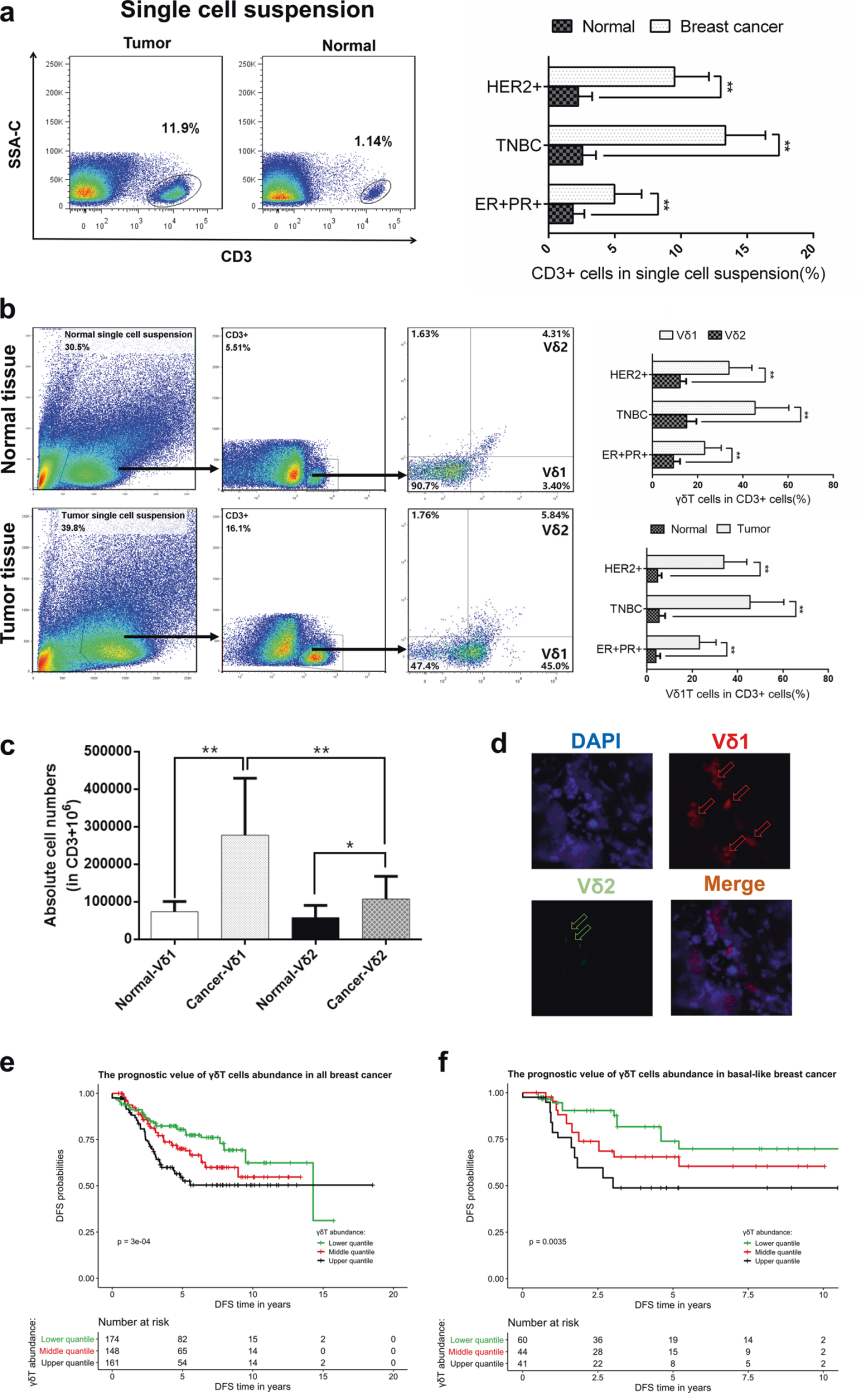
γδ1 T cells make up the majority of tumour-infiltrating lymphocytes (TILs) in breast cancer (BC). **a** Flow cytometry data showing a typical profile of infiltrating CD3+ T cells in paired normal breast tissue and BC specimens. The right bar chart shows the proportion of CD3+ T cells in BC tissue with different molecular subtypes (*n* = 10 of each molecular subtype). **b** Flow cytometry data showing the gating strategy and a typical distribution of Vδ1 and Vδ2 T cells in both normal and tumour tissues. The right bar chart shows their proportions in BC tissues with different molecular subtypes (*n* = 10 per subtype). **c** The absolute numbers of Vδ1 and Vδ2 T cells within the CD3+ T-cell population of normal breast tissue and paired BC specimens were assessed by flow cytometry (*n* = 20). **d** Immunofluorescence data showing typical staining of infiltrating Vδ1 and Vδ2 T cells in frozen BC tissue slices. **e**, **f** Based on the abundance of γδT cells, patients were divided into three groups: lower, middle and upper tertiles, and the disease-free survival (DFS) and overall survival (OS) rates of all BC patients are presented as Kaplan–Meier curves. Data are presented as representative figures or as the means ± SD from independent experiments. *p < 0.05; **p < 0.01

γδ1 T cells were reported to be the dominant infiltrating T cells in BC in a study with a small sample size (ten cases),^3^ but this phenomenon has not been verified in the Chinese population or with different molecular subtypes of BC. Therefore, we examined the prevalence of γδT cells in BC tissue samples with different molecular subtypes (ER+PR+, HER2+ and triple negative, 10 patients per subtype) and paired normal tissue samples. We found that the number of infiltrating lymphocytes was significantly higher in tumour tissues of all molecular subtypes than in the paired normal tissues (*p* < 0.01, Fig. 1a). Moreover, Vδ1+ T cells but not Vδ2+ T cells were revealed to comprise the majority of TILs in all three BC molecular subtypes when gated on CD3 (*p* < 0.01, Fig. 1b, c). In addition, immunofluorescence of frozen sections further confirmed the dominant distribution of Vδ1+ T cells in BC (Fig. 1d).

To further identify the function of breast cancer-infiltrating γδ1 T cells, BC cell lines (MCF-7, T-47D, MDA-MB-231 and MDA-MB-468) were co-cultured with freshly isolated Vδ1+ T cells from breast tumour tissue (E:T ratios: 1:1, 5:1 and 10:1). Upon plotting the cell growth curve, the data show that γδ1 T cells did not exert any inhibitory effect on the growth of BC cells (Supplementary Fig. S1a–d). Next, a cytotoxic assay was performed to compare the killing activity of γδ1 T cells isolated from BC tissues and those isolated from healthy donor peripheral blood (PBHD). PBHD Vδ1+ T cells showed strong killing activity against BC cells, while breast cancer-infiltrating Vδ1+ T cells did not exhibit any killing activity (*p* > 0.05, Supplementary Fig. S2e–h). Since Vδ1+ T cells make up the majority of breast-infiltrating γδT cells, we performed CIBERSORT analysis^11^ using TCGA breast cancer transcriptome and prognostic data sets (GSE6532, GSE9195, GSE16446, GSE17907, GSE19615, GSE20685, GSE20713, GSE21563, GSE31448, GSE42568, GSE48390 and GSE58984) to estimate the abundance of tumour-infiltrating γδT cells. Based on the abundance results, the samples were divided into three groups (high, middle and low), and we found that a high abundance of γδT cells was an unfavourable factor, suggesting a reduction in DFS and OS (Fig. 1e, f). This trend was more prominent in the triple-negative subtype (Supplementary Fig. S2a, b), but no statistically significant difference was observed in luminal breast cancer (data not shown), which might contribute to the lower number of TILs in luminal subtypes. In addition, we also compared the differences in age, T stage and N stage among these three groups (high, middle and low γδT-cell count), and our results indicated a significant correlation between lymph node metastasis and a high abundance of γδT cells (2.20E-16, Supplementary Table S4). Thereafter, to confirm whether γδT cells were an independent factor in prognosis, we performed a multivariate Cox regression and found that a relative abundance of γδT cells served as an independent variable in both OS and DFS (Supplementary Fig. S2, Supplementary Table S4).

### Determination of the phenotype of breast cancer-infiltrating Vδ1+ T cells

**Fig. 2:**
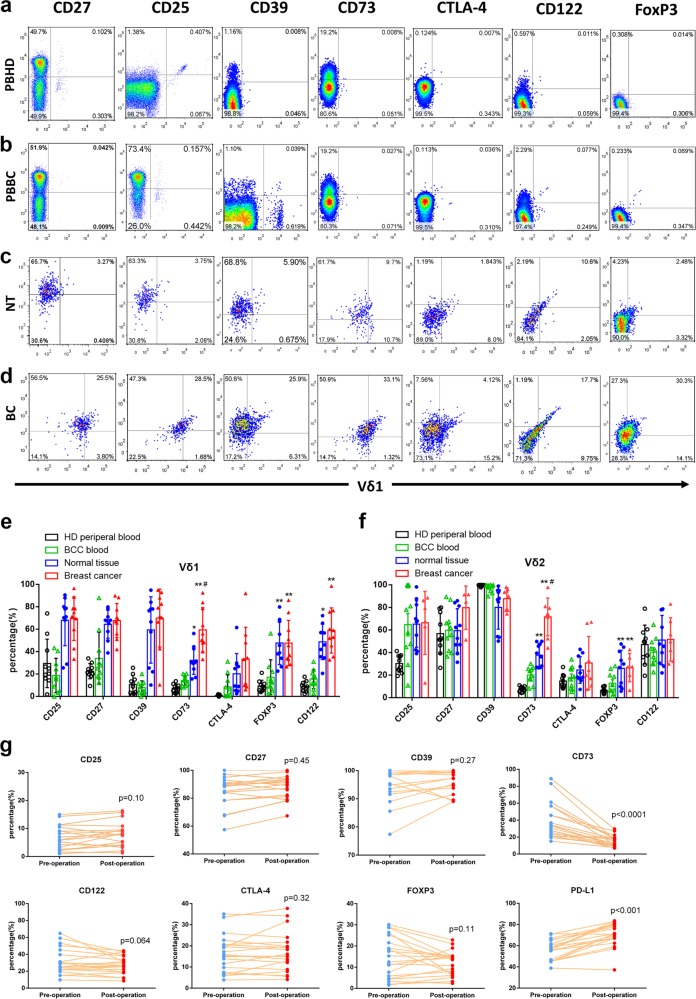
The number of CD73+γδ1 T cells is increased in BC and is related to the tumour burden in peripheral blood (PB). **a** Representative flow cytometry graphs (cells gated on CD3) of the expression of CD25, CD27, CD39, CD73, CD122, CTLA-4 and FoxP3 on CD45+CD3+Vδ1+ T cells, which were isolated from the PB of healthy donors, the PB of breast cancer patients (**b**), normal breast tissue from BC patients (**c**) and breast cancer tissue (**d**). **e**, **f** Bar chart showing the proportion of each subset of CD45+CD3+Vδ1+ and CD45+CD3+Vδ2+ T cells isolated from different origins (*n* = 10). Data are presented as the means ± SD from independent experiments. **p < 0.01 vs PBBC, ^#^*p* < 0.05 vs NT. **g** The expression of CD25, CD27, CD39, CD73, CD122, CTLA-4, FoxP3 and PD-L1 on Vδ1 T cells in PB was analysed by flow cytometry before and 10–14 days after BC surgery (*n* = 20). PB peripheral blood, PBBC peripheral blood from breast cancer patients, PBHD peripheral blood from healthy donors, NT normal tissue, BC breast cancer specimens

Previously, we identified CD39+Vδ T cells as the dominant Tregs in colon cancer.^12^ Here, we aimed to explore whether there is a specific subtype of Vδ1+ T cells in BC that plays a major immunosuppressive role by evaluating the expression of regulatory immune cell markers (CD27, CD25, CD39, CD73, CTLA-4, CD122 and FoxP3) on Vδ1/2+ T cells isolated from PBHDs and Vδ1/2+ T cells isolated from peripheral blood of breast cancer patients (PBBCs), normal breast tissues (NT) and breast cancer (BC) tissues (Fig. 2a–d, Supplementary Fig. S3). Our findings showed that all these Treg markers were increased in infiltrating Vδ1+ T cells from either tumour or normal BC tissue, and only CD73 and FoxP3 expression was increased in infiltrating Vδ2+ T cells. In addition, compared with CD73 expression in Vδ1/2 T cells from PBHDs, CD73 expression in Vδ1/2+ T cells was increased in PBBC, higher in NT and highest in BC (Fig. 2e). Meanwhile, we assessed the levels of these immunosuppressive markers on Vδ1 T cells isolated from PBBCs (20 patients). It is interesting that CD73 expression was obviously decreased 10–14 days after surgery (*p* < 0.0001, Fig. 2g), which indicates that CD73 expression is related to tumour burden. Moreover, PD-L1 levels in Vδ1 T cells were increased post-surgery (*p* < 0.001, Fig. 2g), which may contribute to the post-operative immunosuppressive status.^13^ Furthermore, the expression levels of regulatory immune cell markers (CD27, CD25, CD39, CTLA-4, CD122 and FoxP3) between CD73+ and CD73-Vδ1 T cells in BC tissue were also compared, and the results indicated CD73+ Vδ1 T cells expressed higher levels of CD27, CD122, CTLA-4 and FoxP3 (Supplementary Fig. S3).

We next determined the phenotype of infiltrating Vδ1 T cells by flow cytometry. We found that the expression levels of Granzyme B, Perforin, FasL, TRAIL, Nkp44 and Nkp46 in Vδ1+ T cells were decreased in BC tissues compared with paired normal tissues (Supplementary Fig. S4a, b), and CD73-Vδ1 T cells expressed higher levels of these cytotoxic markers than did CD73+Vδ1 T cells (Supplementary Fig. S4c, d). In addition, cytokine secretion was compared between breast cancer-infiltrating CD73+Vδ1 T and CD73-Vδ1 T cells. The results showed that CD73+Vδ1 T cells expressed significantly higher levels of IL-4, IL-17A, IL-10, GM-CSF and TGF-β than did CD73-Vδ1 T cells (Supplementary Fig. S4e, f).

### CD73+Vδ1 T cells exert immunosuppressive functions and depend on the adenosine-mediated pathway

**Fig. 3:**
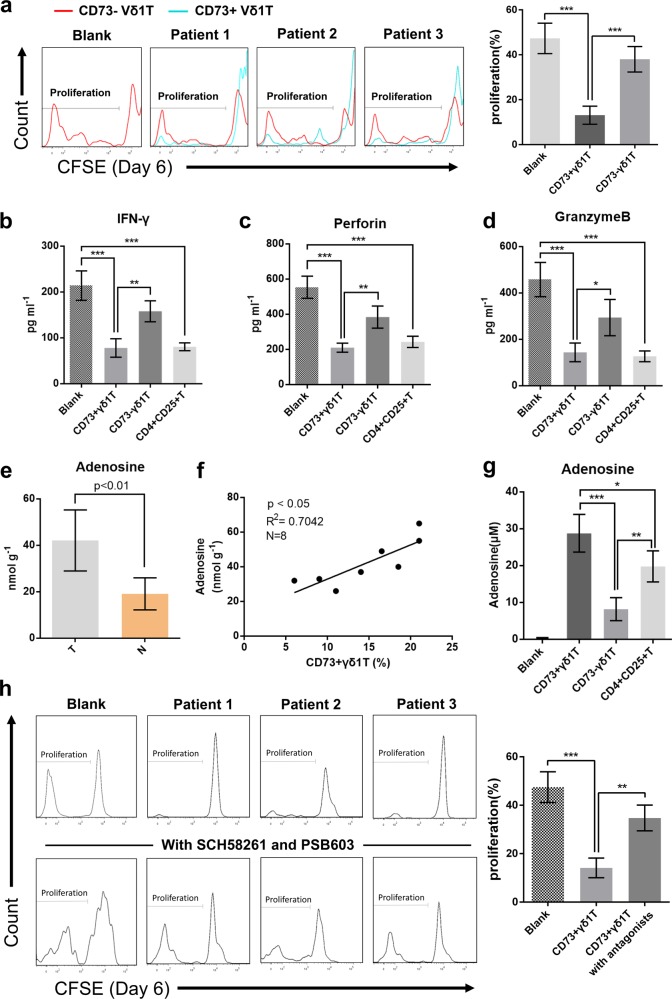
CD73+γδ1 T cells direct exert their immunosuppressive effects via an adenosine-mediated pathway. **a** CD3+CD73-Vδ1 and CD3+CD73+Vδ1 T cells isolated from fresh BC tissues were co-cultured in vitro with CFSE-labelled allogeneic CD3+ T cells in the presence of CD3 and CD28 mAbs, respectively. The proliferation of CD3+ T cells was assessed by flow cytometry on day 6. The right bar chart shows the proliferation ratio (CSFE^low^) of CD3+ T cells co-cultured with CD73-Vδ1 or CD73+Vδ1 T cells. Data are presented as the means ± SD, E:T = 1:1, *n* = 5. **b** CD3+CD73-Vδ1 T cells, CD3+CD73+Vδ1 T cells and CD3+CD4+CD25+ Tregs were isolated from fresh BC tissue and then co-cultured in vitro with allogeneic CD4+ T cells in the presence of CD3 and CD28 mAbs. Concentrations of IFN-γ in the supernatants were detected on day 6 by ELISA. **c**, **d** CD3+CD73-Vδ1 T cells, CD3+CD73+Vδ1 T cells and CD3+CD4+CD25+ Tregs were isolated from fresh BC tissue and then co-cultured in vitro with allogeneic CD8+ T cells in the presence of CD3 and CD28 mAbs. The levels of Perforin and Granzyme B in the supernatants were detected on day 6 by ELISA. Data are presented as the means ± SD, E:T = 1:1, *n* = 5, *p < 0.05, **p < 0.01, ***p < 0.001. **e** Concentrations of adenosine in the supernatants of homogenates from tumour and paired normal tissues were evaluated by fluorometric analysis. Data are presented as the mean ± SD; *n* = 8. **f** Potential correlations of CD73+γδ1 T-cell percentages (among the CD3+ T-cell population) with adenosine concentration in breast tumour tissues (1 g), which was assessed by fluorometric analysis. *N* = 8. **g** CD3+CD73-Vδ1 T cells, CD3+CD73+Vδ1 T cells and CD3+CD4+CD25+ Tregs were isolated from fresh BC tissue and co-cultured with allogeneic CD3+ T cells in the presence of CD3 and CD28 mAbs. The adenosine concentration in the supernatants was evaluated by fluorometric assay on day 6. Data are presented as the means ± SD, E:T = 1:1, *n* = 5, ***p < 0.001. **h** CFSE-labelled CD3+ T cells cultured in the presence or absence of A2A (SCH58261) or A2B (PSB603) antagonists and then co-cultured with CD3+CD73+Vδ1 T cells isolated from breast tumour tissue. CD3+ T-cell proliferation was evaluated on day 6 by flow cytometry. The bar chart summarises the ratio of proliferating CD3+ T cells (CFSE^low^). Data are presented as the means ± SD, *n* = 5, **p < 0.01, ***p < 0.001

To further demonstrate the potent immunosuppressive function of CD73+Vδ1 T cells, we sorted CD73-Vδ1 T and CD73+Vδ1 T cells from fresh BC tissues. As shown in Fig. 3a, the proliferation of CD3+ T cells was greatly inhibited by CD73+, but not CD73-Vδ1 T cells (*p* < 0.001). In addition, similar to CD4+CD25+ Tregs, we found that tumour-infiltrating CD73+Vδ1 T cells could suppress IFN-γ secretion from CD4+ T cells (Fig. 3b) and Perforin and Granzyme B secretion from CD8+ T cells (Fig. 3c, d). Taken together, these data suggest that CD73+Vδ1 T cells are the predominant Tregs in human BC not only in quantity but also in effectiveness.

Previous studies have shown that CD39 and CD73 are ectonucleotidases that hydrolyse extracellular ATP to adenosine, a classic and very strong immunosuppressive agent.^14^ Similar to our previous results in colon cancer,^12^ the level of adenosine in breast tumour tissues was much higher than that in normal tissues (*p* < 0.01, Fig. 3e). Surprisingly, we also found a positive correlation between adenosine levels and the CD73+Vδ1 T-cell ratio (*R*^2^ = 0.70, *p* < 0.05, Fig. 3f). We then evaluated the levels extracellular adenosine from the supernatants of different breast cancer-infiltrating immune cells (CD73+Vδ1 T, CD73-Vδ1 T and CD4+CD25+ T cells) co-cultured with CD3+ T cells and found that adenosine was highest in the presence of CD73+Vδ1 T cells compared with that in the presence of the other cell types (Fig. 3g). To confirm whether CD73+Vδ1 T cells exert immunosuppressive function through the adenosine pathway, a proliferation assay was performed in the presence or absence of the A2A adenosine receptor antagonist SCH58261 and A2B antagonist PSB603. The results showed that CD3+ T-cell proliferation was almost derepressed by the addition of SCH58261 and PSB603 (Fig. 3h). In addition, because high levels of IL-10 and TGF-β were found in CD73+Vδ1 T cells (Supplementary Fig. S3e, f) and these two cytokines are involved in the immunosuppressive function of Tregs,^15^ we added IL-10- and TGF-β-neutralising antibodies into the co-culture system, but the inhibitory effect of CD73+Vδ1 T cells was not reversed (Supplementary Fig. S5a, b). Overall, these data indicate that the CD73+Vδ1 T-cell-mediated immunosuppressive effects mainly rely on the adenosine pathway.

### Breast cancer cells induce CD73 expression in Vδ1 T cells via exosomes and the TGF-β pathway

**Fig. 4:**
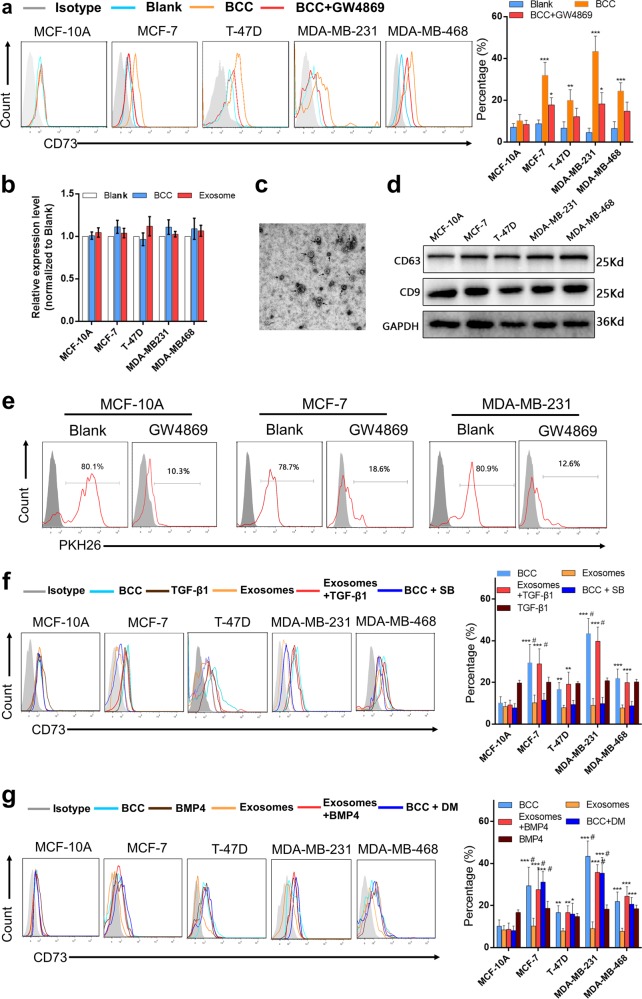
BCCs induce CD73 expression in γδ1 T cells, which is dependent on exosomes and TGF-β1. **a** CD3+Vδ1 T cells sorted from PBHDs and co-cultured with BCCs (control or pretreated with 10 μm GW4869 for 24 h) were incubated in transwells for 24 h. CD73 expression in Vδ1 T cells was determined by flow cytometry. The right bar chart shows the proportion of the CD73+ subsets; data are shown as the means ± SD, *n* = 5, vs blank *p < 0.05, **p < 0.01. **b** CD3+Vδ1 T cells sorted from PBHDs were co-cultured with different BCCs in transwells or incubated with exosomes. The mRNA level of CD73 was evaluated by RT-PCR, *n* = 5. **c** Representative transmission electron micrograph of exosomes isolated from the supernatants of BC cell lines. **d** Western blot analysis of the exosome markers CD63 and CD9 in exosomes shed from different BC cell lines. **e** Exosomes isolated from the supernatants of BCCs (NC or pretreated with 10 μm GW4869 for 24 h), stained with PKH26, and then co-cultured for 24 h with Vδ1 T cells isolated from PBHDs. Then, the internalisation of exosomes was determined by flow cytometry. **f** CD3+Vδ1 T cells sorted from PBHDs and then co-cultured with BCCs or their exosomes (in the presence or absence of TGF-β1 or SB-431542 pretreatment) were incubated in transwells for 24 h. TGF-β1 treatment alone was performed as a control. CD73 expression in Vδ1 T cells was determined by flow cytometry. The right bar chart shows the CD73+ proportion of cell subsets. **g** CD3+Vδ1 T cells sorted from PBHDs and then co-cultured with BCCs or their exosomes (in the presence or absence of BMP4 or dorsomorphin pretreatment) were incubated in transwells for 24 h. BMP4 treatment alone was performed as a control. CD73 expression in Vδ1 T cells was determined by flow cytometry. The right bar chart shows the CD73+ proportion of cell subsets. Data are shown as the means ± SD, *n* = 5. vs MCF-10A, *p < 0.05, **p < 0.01, ***p < 0.001; vs TGF-β1 or BMP4, ^#^*p* < 0.05. BCC breast cancer cell, PBHDs peripheral blood of healthy donors

According to the above results and a previous report that breast cancer-infiltrating γδT cells are recruited from peripheral blood^4^, we speculated that BCCs mainly promote the CD73 phenotypic transformation of Vδ1 T cells in a non-contact manner. Increasing studies have suggested that tumour cells regulate the immune microenvironment via exosomes; therefore, Vδ1 T cells isolated from PBHDs were co-cultured with different BCCs in transwells, some of which were pretreated with GW4869 (10 μm, 24 h), which can inhibit exosome secretion. Our findings revealed that the levels of Vδ1 T cells were enhanced by co-culture with BCCs in transwells, while this effect was significantly suppressed in the presence of GW4869 (Fig. 4a). Moreover, the CD73 mRNA level in Vδ1 T cells was also detected, and the negative result suggested that a post-transcriptional regulatory mechanism was involved (Fig. 4b). Thereafter, exosomes were isolated from the supernatant of BCCs, and their typical signatures were identified via transmission electron microscopy and western blotting of CD9 and CD63 (Fig. 4c, d). Then, breast TDEs were collected and labelled with PKH26 (Fig. 4e), and flow cytometry analysis proved that TDEs could be taken up by Vδ1 T cells isolated from PBHDs. Furthermore, Vδ1 T cells were co-cultured with TDEs, but the results showed that TDEs alone could not promote CD73 expression in Vδ1 T cells (Fig. 4f, g).

Previous reports imply that several transcription factors, such as SMAD3/5, can bind to the promoter region of CD73;^16^ therefore, we hypothesised that TGF-β1 or BMP4, both of which are highly expressed in the tumour microenvironment, may function as a catalyst in this interaction. To confirm this, we repeated the co-culture assay in the presence of TGF-β1 (10 ng/ml) or BMP4 (10 ng/ml), and cells were pretreated with SB-431542 (10 μm, 1 h) or dorsomorphin (10 μm, 1 h). Surprisingly, TDEs plus either TGF-β1 or BMP4 obviously promoted CD73 expression in Vδ1 T cells, but only the TGF-β1 receptor inhibitor SB-431542 blocked CD73 upregulation in the BCC co-cultures (Fig. 4f, g). Classically, TGF-β1 signals are transduced via the TGFRB2 and TGFRB1 receptors (ALK5) to activate SMAD2/3, while BMP4 transduces signals via type I receptors (ALK1, -2, -3 and -6) to activate SMAD1/5/8.^17^ However, noncanonical signals linking TGF-β1 to SMAD1/5 have been described in epithelial and malignant cells.^18,19^ Therefore, the above experimental results suggest that crosstalk between TGF-β1 and SMAD1/5 and between TDEs and TGF-β1/SMAD pathway activation collaborate to enhance CD73 expression in Vδ1 T cells.

### TDEs transfer lncRNA SNHG16 to enhance SMAD5 expression in Vδ1 T cells

**Fig. 5:**
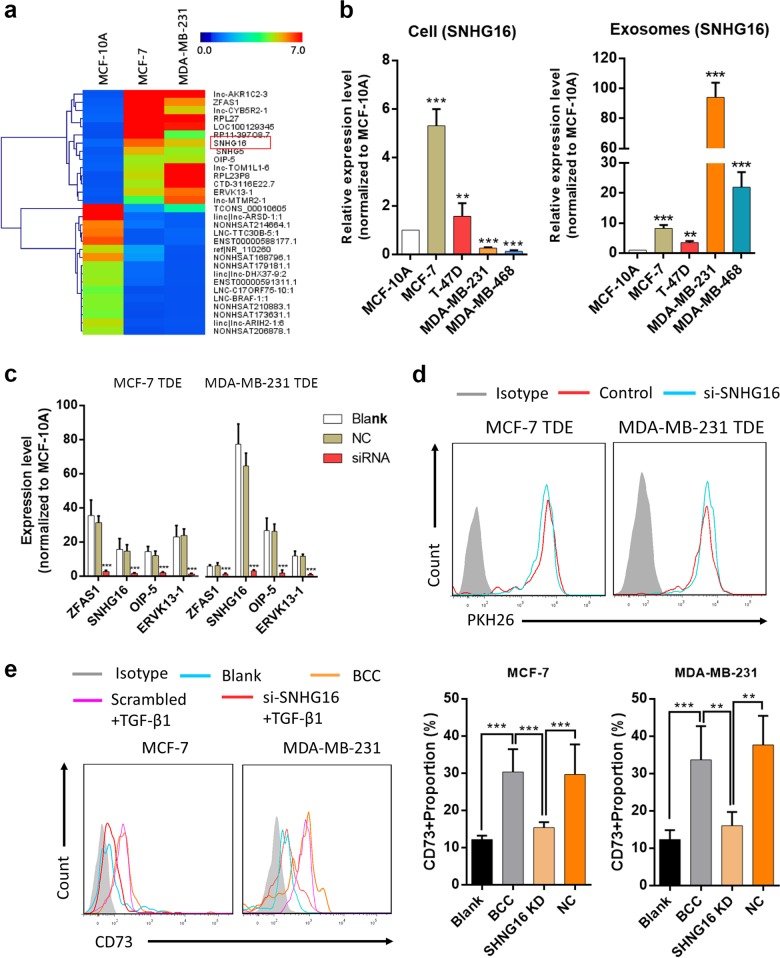
Tumour cell-derived exosomes (TDEs) upregulate CD73 expression in γδ1 T cells through the long non-coding RNA (lncRNA) SNHG16. **a** Heatmap showing the common up- or downregulated lncRNAs in TDEs (MCF-7 cells/MDA-MB-231 cells vs MCF-10A cells), where red and blue denote high and low levels of expression, respectively. **b** SNHG16 expression in BCCs (MCF-10A, MCF-7, T-47D, MDA-MB-231 and MDA-MB-468) and their corresponding exosomes was evaluated by RT-PCR. The data are shown as the means ± SD, *n* = 3, vs MCF-10A **p < 0.01, ***p < 0.001. **c** BCCs were transfected with different target siRNAs or control siRNA, and the expression levels of exosomal lncRNAs were then evaluated with RT-PCR. The data are shown as the means ± SD, *n* = 3, vs blank ***p < 0.001. **d** Representative graphs of PKH26-stained exosomes isolated from the supernatants of MCF-7, si-SNHG16-MCF-7, MDA-MB-231 and si-SNHG16-MDA-MB-231 cells and co-cultured for 24 h with Vδ1 T cells, which were isolated from PBHDs. The internalisation of exosomes was determined by flow cytometry. **e** MDA-MB-231 and MCF-7 cells were transfected with control siRNA or si-SNHG16, and then the cancer cells or their exosomes were collected and co-cultured with Vδ1 T cells (isolated from PBHDs) for 24 h in the presence of TGF-β1. CD73 expression on Vδ1 T cells was determined by flow cytometry. Data are shown as the means ± SD, *n* = 4, **p < 0.01, ***p < 0.001. BCC breast cancer cell, PBHDs peripheral blood of healthy donors

Exosomes are enriched in non-coding RNAs and are considered important vehicles to regulate the immune microenvironment.^20^ Because of the positive regulatory effect of TDEs on CD73 expression in Vδ1 T cells and because lncRNAs have been reported to regulate the phenotype of immune cells,^21,22^ a microarray analysis was performed with exosomes derived from MCF-10A, MCF-7 and MDA-MB-231 cells to identify differentially expressed lncRNAs. We found that in comparison with those in exosomes derived from MCF-10A cells, 14 lncRNAs from TDEs (MCF-7 and MDA-MB-231) simultaneously showed higher expression (≥fourfold vs. MCF-10A-derived exosomes, Fig. 5a). Then, we isolated exosomes by ultracentrifugation, and RT-PCR confirmed that four lncRNAs (SNHG16, ZFAS1, OIP-5 AS1 and ERVK3–1) were significantly increased in TDEs (Supplementary Fig. S6a). Moreover, we found that the expression levels of these genes were not identical between exosomes and cells (Fig. 5b; Supplementary Fig. S6b–d), which has also been addressed in other studies.^23^ Afterwards, BCCs were transfected with lncRNA-specific siRNAs, and RT-PCR confirmed that the expression of these lncRNAs was also inhibited within exosomes (Fig. 5c). Then, TDEs were isolated and co-cultured with Vδ1 T cells, and our findings indicated that with the exception of SNHG16, none of the other three lncRNAs could modulate CD73 expression (Fig. 5d, e; Supplementary Fig. S6e–g), demonstrating that the TDEs induce CD73 expression in Vδ1 T cells by delivering lncRNA SNHG16.

Previous results revealed that both TDEs and the TGF-β/SMAD pathway participate in the regulation of CD73+Vδ1 T cells, so we evaluated the expression of total SMAD2, SMAD3, SMAD1 and SMAD5 in Vδ1 T cells after co-culture with TDEs. First, TDEs were isolated and co-cultured with Vδ1 T cells, and the protein expression of total SMADs was measured by flow cytometry. Our results revealed that SMAD5 levels increased significantly, while those of SMAD1/2/3 remained unchanged. Thereafter, we isolated TDEs from MDA-MB-231 and MCF-7 cells transfected with SNHG16 siRNA and repeated the co-culture experiment, which showed that the SMAD5 level was no longer upregulated (Supplementary Fig. S7a, b). Furthermore, the activation of TGF-β1/SMAD5 was also evaluated. We found co-culturing with TDEs derived from BCCs greatly upregulated the level of p-SMAD5 in Vδ1 T cells, and that inhibiting exosome secretion with GW4869 significantly decreased the p-SMAD5 and CD73 levels (Supplementary Fig. S7c, d). In addition, Vδ1 T cells with or without SB-431542 (10 μm, 1 h) or dorsomorphin (10 μm, 1 h) pretreatment were co-cultured with TDEs in the presence of TGF-β1. Our results indicated that the levels of p-SMAD5 and CD73 could not be modulated by TDEs alone, but when combined with TGF-β1, the TDEs increased the levels of p-SMAD5 and CD73, and this effect could be suppressed by SB-431542 but not dorsomorphin (Supplementary Fig. S7e, f). In conclusion, TDEs promoted the expression of SMAD5 in Vδ1 T cells by transferring lncRNA SNHG16, which further potentiated the TGF-β1/SMAD5 pathway and resulted in CD73 upregulation.

### SNHG16 sponges miR-16–5p to upregulate the expression of SMAD5

**Fig. 6:**
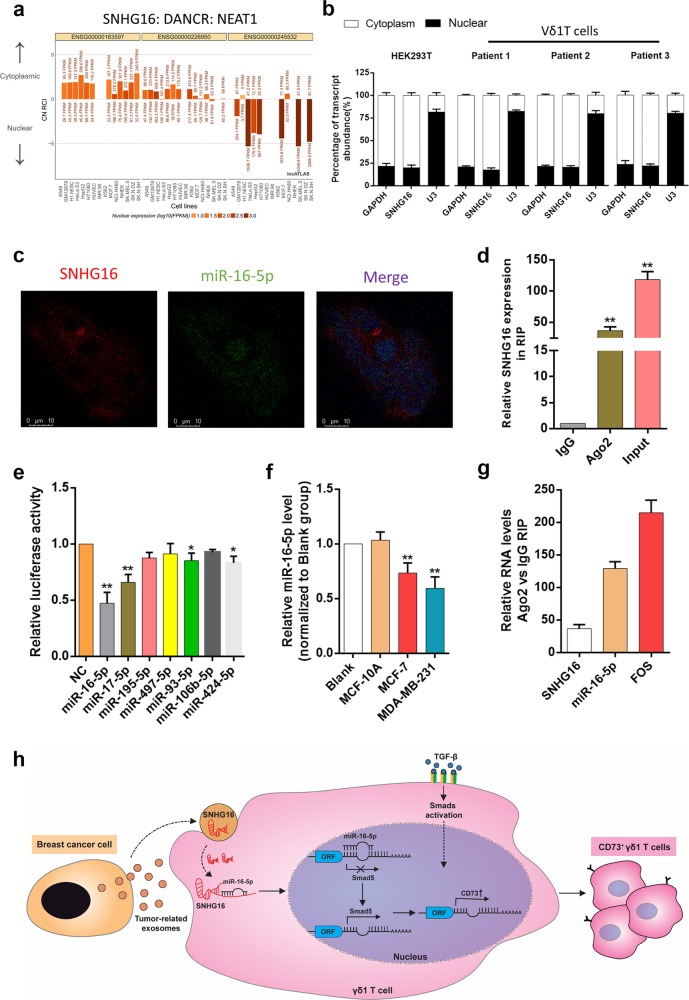
SNHG16 is located in the cytoplasm and functions as a ceRNA by sponging miR-16-5p. **a** SNHG16 was predicted by the bioinformatics tools in lncATLAS to be located mainly in the cytoplasm. **b** Quantitative RT-PCR analysis of subcellular SNHG16 expression in the nucleus and cytoplasm of HEK293T and Vδ1 T cells isolated from PBHDs. **c** RNA fluorescence in situ hybridisation (FISH) shows the location of SNHG16 and miR-16-5p in HEK293T cells. **d** Vδ1 T cells were co-cultured with exosomes isolated from BCCs, and the fold enrichment of SNHG16 was assessed by an AGO2-RIP assay. Data are shown as the means ± SD, *n* = 3, vs IgG **p < 0.01. **e** Luciferase activity was evaluated by co-transfection of reporter plasmid (pGLO-SNHG16) and seven various miRNA mimics or miR-control into HEK293T cells. The data are shown as the means ± SD, *n* = 3, vs NC *p < 0.05, **p < 0.01. **f** Vδ1 T cells were co-cultured with exosomes isolated from MCF-10A, MDA-MB-231 and MCF-7 cells for 24 h, and the expression level of miR-16-5p was determined by RT-PCR. Data are shown as the means ± SD, *n* = 3, vs blank **p < 0.01. **g** RNA expression levels in Ago2 immunoprecipitates were evaluated as fold enrichment relative to those in IgG immunoprecipitates. **h** Schematic illustrating the mechanism by which breast TDEs upregulate CD73 expression in tumour-infiltrating Vδ1 T cells, with SNHG16 competitively binding miR-16-5p to upregulate the activating ability of the TGF-β1/SMAD5 pathway. Data are shown as the means ± SD, *n* = 3; PBHD peripheral blood of healthy donors

To further elucidate the mechanism by which SNHG16 promotes SMAD5 expression, we used lncATLAS (http://lncatlas.crg.eu/) to predict the subcellular localisation of SNHG16. The results showed that SNHG16 was predicted to be mainly located in the cytoplasm of all available cell lines (Fig. 6a). Next, quantitative RT-PCR analysis verified that SNHG16 was localised mainly in the cytoplasm of HEK293T and Vδ1 T cells (Fig. 6b), which was also confirmed by FISH (Fig. 6c). Based on the above results, we assumed that SNHG16 acts as a competitive endogenous RNA (ceRNA) here. Ago2 is recognised as the core effector protein of the RNA-induced silencing complex (RISC), which is involved in miRNA-mediated mRNA destabilization or translational repression; therefore, an RIP assay was performed with anti-Ago2 antibody. Our results showed that SNHG16 was enriched in Ago2-RIPs compared with control IgG-RIPs (Fig. 6d, *p* < 0.01), which indicates that SNHG16 acts as a ceRNA to upregulate CD73 expression in Vδ1 T cells.

Next, we applied bioinformatic analysis with the DIANA, starBase and TargetScan databases to predict the miRNAs that could both bind SNHG16 and the 3’UTR of SMAD5 and selected seven commonly predicted miRNAs for further analysis (Fig. S7a). Using a dual-luciferase assay, we observed that miR-16–5p, miR-17–5p, miR-93–5p and miR-424–5p decreased the luciferase activity of pmirGLO-SNHG16, indicating that these miRNAs could directly bind to SNHG16 through their respective miRNA-binding sites (Fig. 6e). Next, different TDEs were co-cultured with Vδ1 T cells, and the expression levels of the above miRNAs were assessed by RT-PCR. Our results showed that the expression of miR-17–5p, miR-424–5p and miR-93–5p was almost unchanged (Supplementary Fig. S7c), but that of miR-16–5p was significantly decreased (Fig. 6f). The RIP assay found that SNHG16 and miR-16–5p were both enriched in the Ago2 immunoprecipitates of Ago2 compared with the control IgG immunoprecipitates (Fig. 6g). In addition, in HEK293T cells, SNHG16 expression was obviously suppressed by miR-16–5 overexpression and increased by miR-16–5p silencing (Supplementary Fig. S9a, *p* < 0.01), whereas SNHG16 overexpression significantly decreased miR-16–5p expression (Supplementary Fig. S9b, *p* < 0.01). These results proved that SNHG16 directly “sponges” miR-16–5p.

Finally, we needed to verify that SMAD5 is a miR-16–5p target gene. We constructed the SNHG16-miR-16–5p-targeted ceRNA network in BC using TCGA data and found that SMAD5 might be involved in this network (Supplementary Fig. S7b). We transfected miR-16–5p siRNA and miR-16–5p mimics into the HEK293T, MDA-MB-231 and MCF-7 cell lines. SMAD5 expression was determined by western blot, and our results showed that SMAD5 expression could be regulated by miR-16–5p (Supplementary Fig. S10a). Luciferase reporter assays showed that overexpression of miR-16–5p could repress the luciferase activity of cells transfected with the wild-type SMAD5 3′-UTR reporter plasmid, while no obvious inhibition was observed in cells transfected with the mutant reporter plasmid (Supplementary Fig. S10b–c). Furthermore, we also tried to confirm that manipulating miR-16–5p could affect the expression of both SMAD5 and CD73 in γδ1 T cells. Upon transient transfection, we found that SMAD5 expression was affected by miR-16–5p upregulation or inhibition (Supplementary Fig. S10d). Transient transfected γδ1 T cells (transfected with miR-16–5p mimics or inhibitor) were co-cultured with si-SNHG16-MDA-MB-231 cells, and we found that miR-16–5p inhibition in Vδ1 T cells could affect CD73 expression (Supplementary Fig. S10e). Taken together, these results reveal the existence of an exosomal SNHG16/miR-16–5p/SMAD5-regulatory axis, which potentiates the activation of the TGF-β1/SMAD5 pathway and thus induces CD73 expression in Vδ1 T cells (Fig. 6h).

## Discussion

In this study, we found that infiltrating γδ1 T cells are greatly increased in BC and identified CD73+γδ1 T cells as novel regulatory T cells in BC. Tumour-infiltrating CD73+γδ1 T cells express high levels of IL-4, IL-17A, IL-10, GM-CSF and TGF-β, and exert immunosuppressive functions mainly via the adenosine-mediated pathway. Our further research revealed that breast TDEs are responsible for the induction of CD73+γδ1 T cells, which is dependent on the TGF-β1/lncRNA SNHG16/miR-16–5p/SMAD5 pathway.

Although γδT cells account for only 0.5–5% of all T lymphocytes, their immunoregulatory effect in the tumour microenvironment has garnered increasing attention in recent years. Previously, it was widely believed that γδT cells functioned as antagonists in cancer development, but recent research indicates that γδT cells could be polarised to other phenotypes to suppress antitumour immunity. Vδ2 γδ T cells were found to be polarised towards FOXP3+ γδ Tregs upon stimulation with TGF-β and IL-15 in vitro and inhibited the proliferation of PBMCs.^24^ In addition to Vδ2 γδ T cells, Vδ1 γδ T cells have a more potent regulatory potential than αβ Tregs (CD4+CD25+).^12^ Previous studies indicated that γδ1 T cells are the major immunosuppressive T-cell type in human BC,^3,4^ which we also observed here, but it is still not rational to eradicate the whole γδ1 T population owing to its important role in both innate and acquired immunity.^25^ Here, we unexpectedly discovered that CD73+γδ1 T cells are the major Tregs in BC and exert immunosuppressive functions via the adenosine pathway. Upon comparing CD73+ and CD73-γδ1 T cells that were freshly isolated from human BC tissues, we first showed that the CD73+ subpopulation had greater immunosuppressive capacity to inhibit CD3+ T-cell proliferation and CD4+ and CD8+ T-cell function.

The presence of high levels of extracellular adenosine in tumours has been proven to play a crucial role in the evasion of antitumour immunity.^26^ Extracellular adenosine is generated by two hydrolysis steps: first, ectonucleoside triphosphate diphosphohydrolase-1 (ENTPD1), also referred to as CD39, reversibly hydrolyses ATP into AMP; then, CD73 irreversibly hydrolyses extracellular AMP into adenosine and inorganic phosphate.^14^ In addition, increasing evidence has indicated that CD73 expression is upregulated and usually co-localises with CD39 in tumour-infiltrating immunosuppressive cells, such as Tregs (CD4+CD25+FoxP3+), the M2 subset of macrophages and MDSCs,^27–30^ which results in the generation of more adenosine and the suppression of other immune cells expressing A2A adenosine receptors. Recently, Neo et al.^31^ found that BCCs could induce CD73 expression in infiltrating NK cells upon engagement of 4–1BBL on tumour cells, which facilitated tumour cell escape from immunity. Based on accumulating preclinical evidence, the therapeutic potential of targeting the CD73/A2AR axis in solid tumours has been tested in preclinical experiments or clinical trials:^32^ (1) targeting the soluble form of CD39 and CD73 efficiently promotes antitumour immunity and exhibits a synergistic effect with oxaliplatin,^33^ and (2) the use of anti-CD73 mAb or small inhibitory molecules either as a single agent or combined with anti-PD-L1 antibody have revealed good tolerance similar to anti-PD-L1 monotherapy and encouraging immunological response.^34,35^ In this study, we report for the first time that the γδT population with high CD73 expression co-expresses CD39 and exerts an immunosuppressive effect in an adenosine-dependent manner.

CD73 expression on immune cells has been found to be related to the TGF-β/SMAD pathway. TGF-β1 can induce CD73 expression in murine CD4+Foxp3-T cells, CD8+ T cells^36,37^ and MDSCs.^38^ In addition, the expression of CD73 can be modulated by the following post-transcriptional mechanism: miR-223 and miR-23b suppress the expression of SP1,^39,40^ and miR-200c and miR-142–5p suppress the expression of SMAD2/3,^41,42^ both of which can indirectly decrease CD73 levels. Here, our results revealed that CD73 expression in γδ1 T cells was also modulated by the TGF-β1/SMAD pathway, but, unexpectedly, TDEs greatly participated in this process; therefore, we attempted to uncover the detailed mechanism.

Within the tumour microenvironment, exosomes are considered vehicles for information transfer between cancer cells and immune cells. Unlike cellular content, most functional nucleic acids within exosomes are non-coding RNAs, including miRNA, lncRNA and circRNA.^9,20^ We found that inhibiting exosome release from BCCs greatly impaired the upregulation of CD73 expression in γδ1 T cells, implying that exosomes play a pivotal role in this biological process. Recent studies have highlighted TDEs as important contributors to the immunosuppressive tumour microenvironment by transferring non-coding RNAs, which include miRNAs and lncRNAs, to immune cells; these phenotypes include inducing a suppressor phenotype or even apoptosis in CD8+ T cells^43,44^ and regulating macrophage M1/M2 polarisation.^21,22,45^ LncRNA SNHG16 has been reported to be an indicator of poor prognosis in malignant diseases,^46,47^ and has been revealed to both play a role as a ceRNA and be involved in the regulation of the JAK/STAT3, WNT/beta-catenin and SMAD pathways.^48–50^ We are the first group to report that SNHG16 can be transferred into tumour-infiltrating γδ1 T cells via TDEs and is essential for CD73 upregulation. Although it is difficult to perform gain- and loss-of-function experiments in primary human γδ1 T cells to prove the SNHG16/miR-16–5p/SMAD5-regulatory axis, the RIP assay indicated that SNHG16 sponges miR-16–5p in γδ1 T cells, and in vitro experiments identified SMAD5 as a direct target of miR-16–5p.

In summary, our study revealed infiltrating CD73+γδ1 T cells as a key immunosuppressive component in the BC microenvironment, and we speculated that BCC-derived exosomal SNHG16 promotes the activation of the TGF-β1/SMAD5 pathway by sponging miR-16–5p and results in the conversion of γδ1 T cells into the CD73+ immunosuppressive subtype. It is worth noting that some in vitro studies are correlative and descriptive due to the nature of the human studies. Nevertheless, our results suggest the significance of this subpopulation, and targeted therapy against these cells might have potential for BC treatment in the future.

## Supplementary information

Supplementary Materials

## Data Availability

The data sets used and/or analysed during the current study are available from the corresponding author on reasonable request.
