# Supplementary material for: Breast cancer-derived exosomes transmit lncRNA SNHG16 to induce CD73+γδ1 Treg cells
Source: Signal Transduct Target Ther. 2020 Apr 29;5:41. doi: 10.1038/s41392-020-0129-7 (PMC7188864; doi:10.1038/s41392-020-0129-7)
Supplement: Supplementary file 1 — Supplementary Materials [file 41392_2020_129_MOESM1_ESM.docx]

Supplementary Materials for

**Breast cancer-derived exosomes transmit lncRNA SNHG16 to induce CD73+γδ1 Treg cells**

Chao Ni ^#^*, Qing-Qing Fang^#^, Wu-Zhen Chen^#^, Jing-Xing Jiang, Zhou Jiang, Jun Ye, Ting Zhang, Liu Yang, Fan-Bo Meng, Wen-Jie Xia, Miaochun Zhong, Jian Huang*

Correspondence to: [nicaho428@zju.edu.cn](mailto:nicaho428@zju.edu.cn); [drhuangjian@zju.edu.cn](mailto:drhuangjian@zju.edu.cn).

**This PDF file includes:**

Figures. S1 to S10

Tables S1 to S4


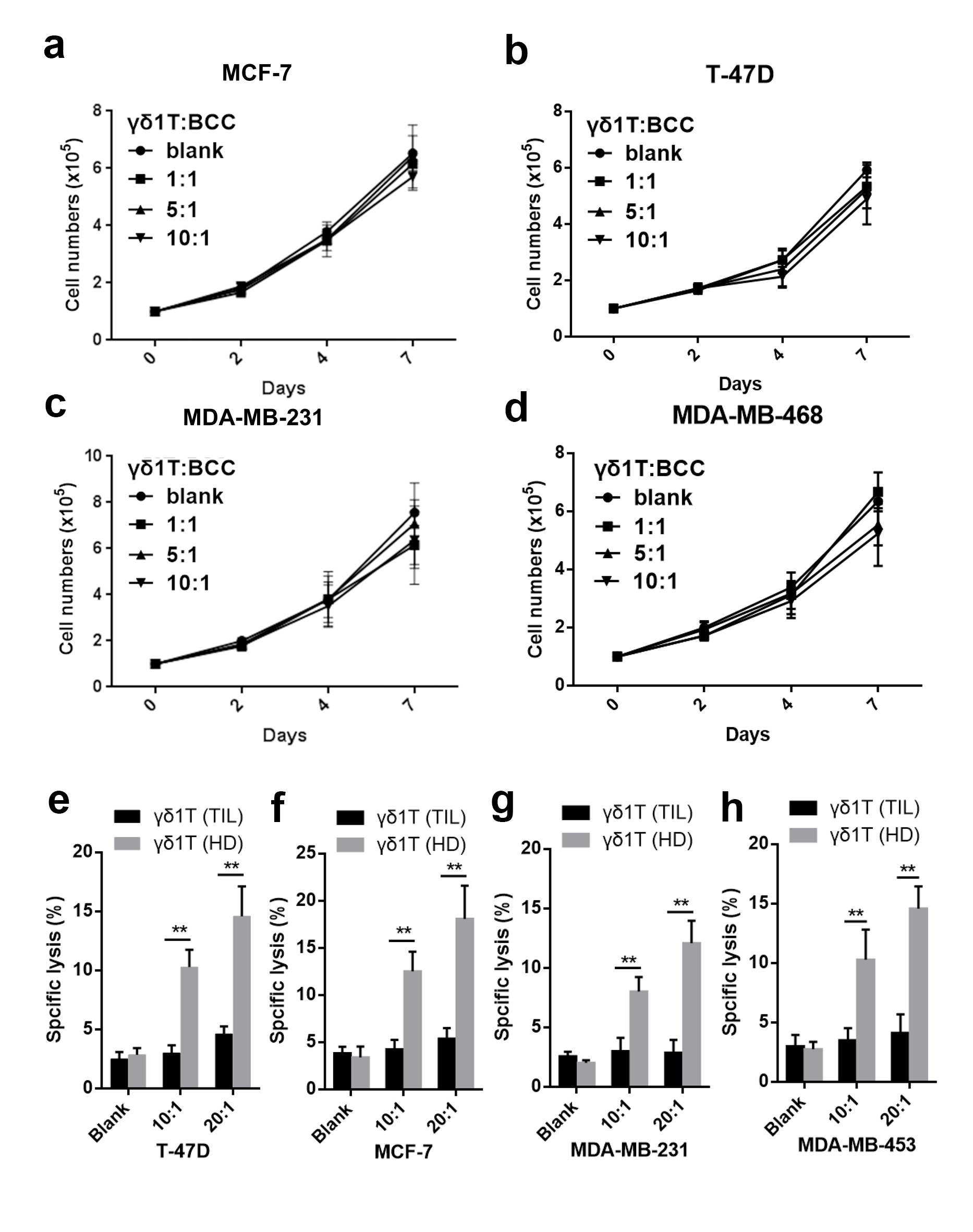


Figure. S1. Cytotoxicity of γδ1 T cells freshly isolated from BC tissue and PB.


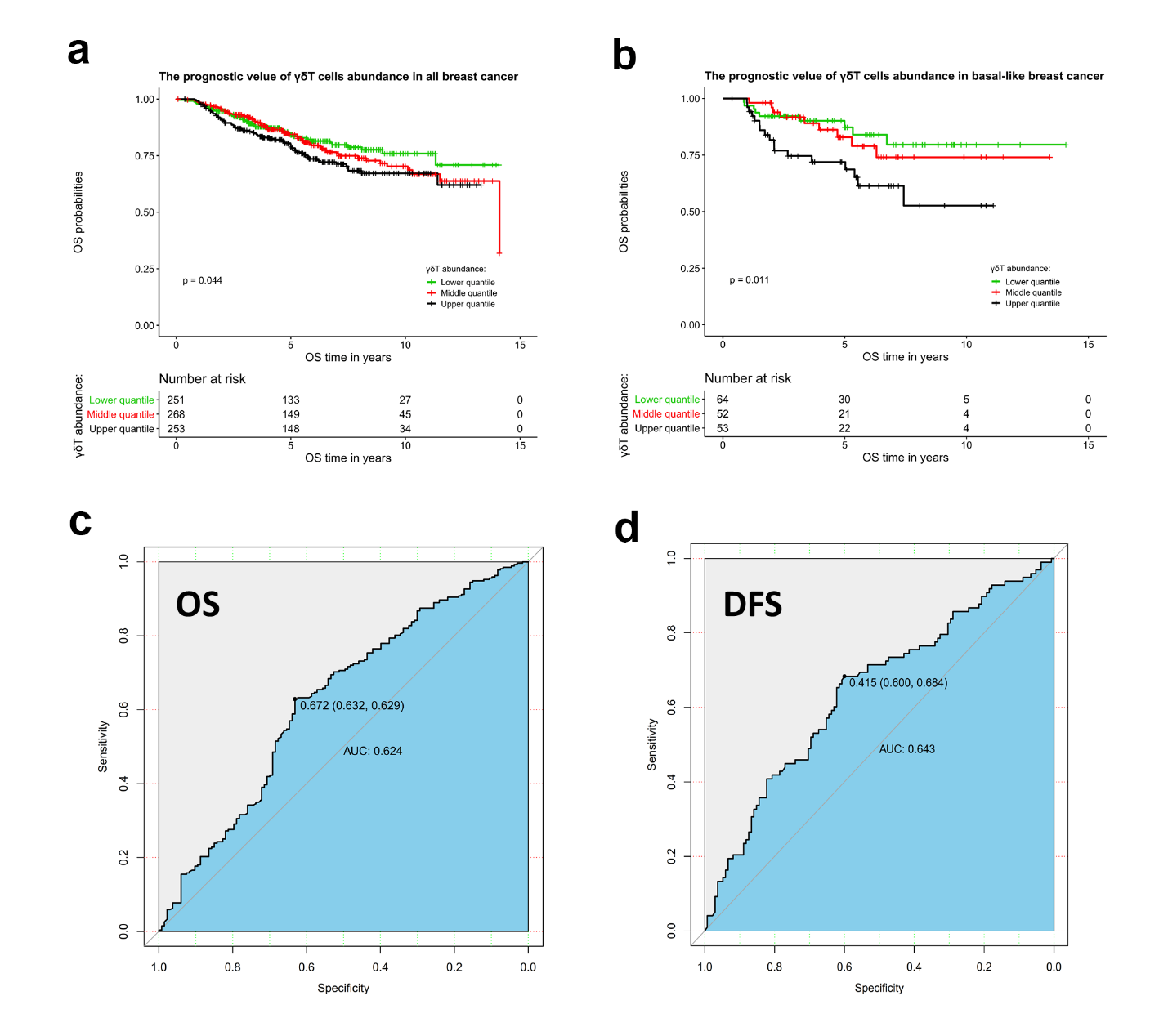


**Figure. S2.** Prognostic value of breast cancer-infiltrating γδT cells analysed with CIBERSORT and multivariate Cox regression analysis.


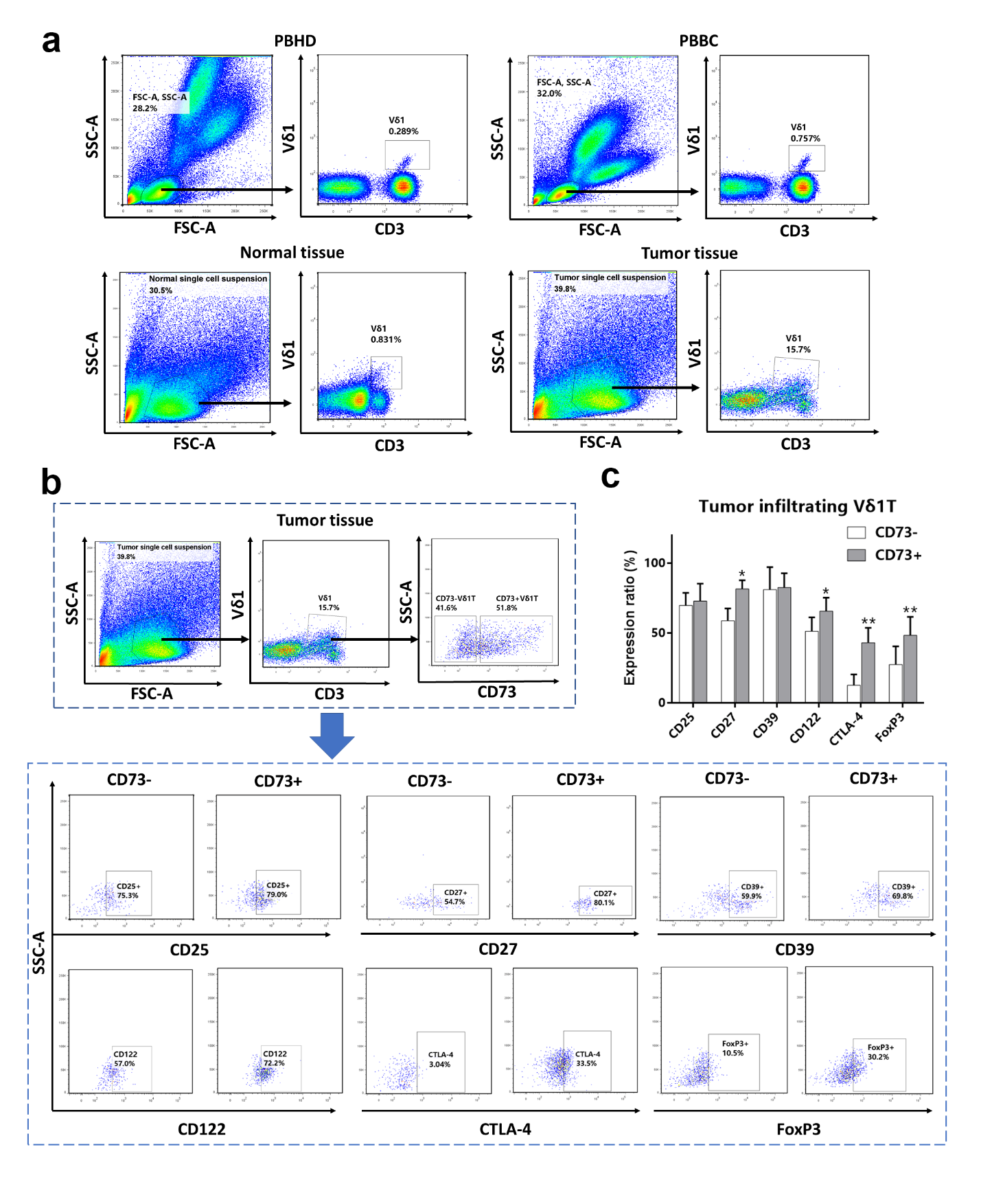


Figure. S3. Gating strategy of Vδ1 T cells within different samples and the expression levels of regulatory immune cell markers on CD73+ and CD73-γδ1T cells.


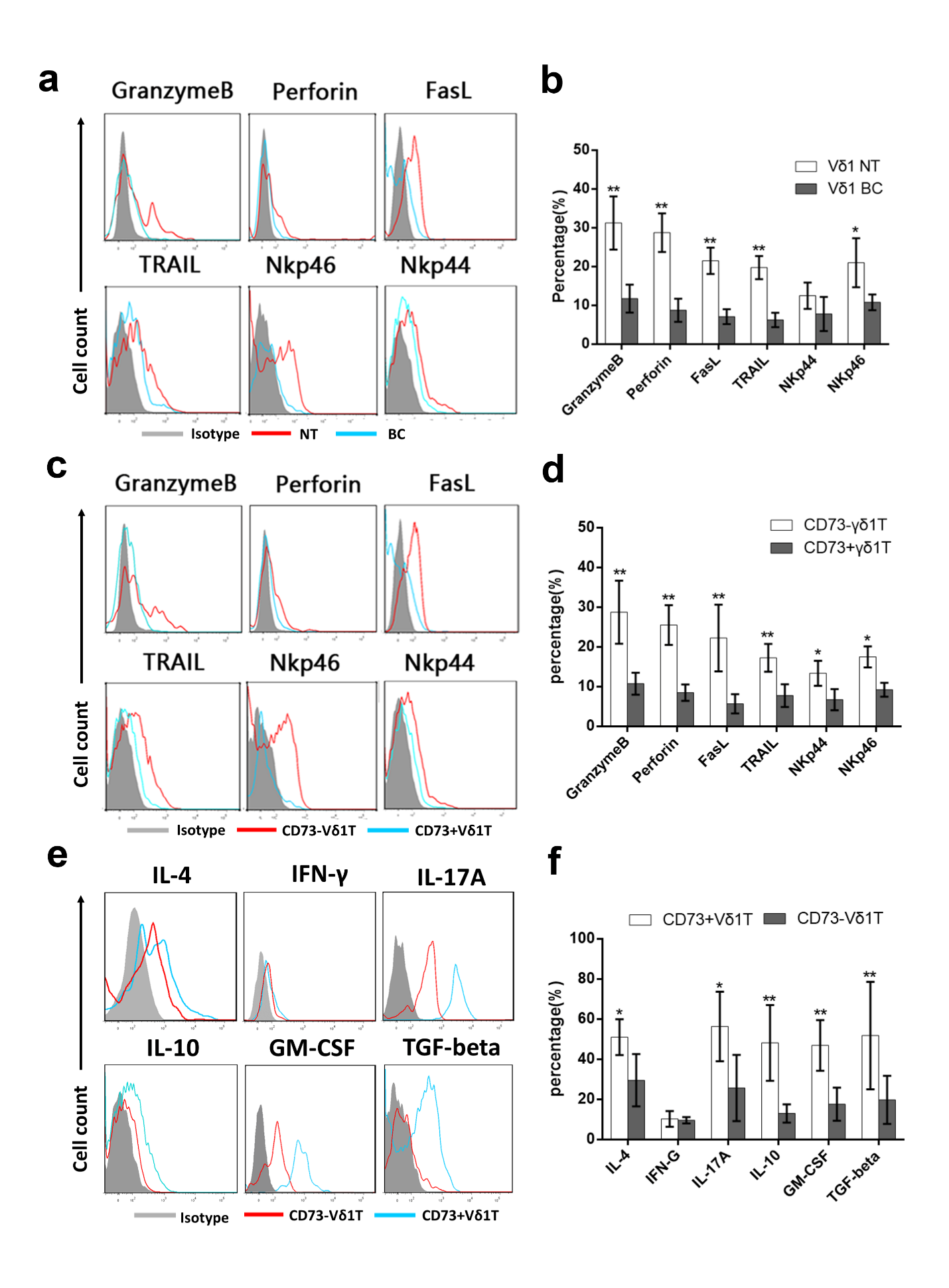
Figure. S4. Characteristics of γδT cells in BC specimens and paired normal tissues.


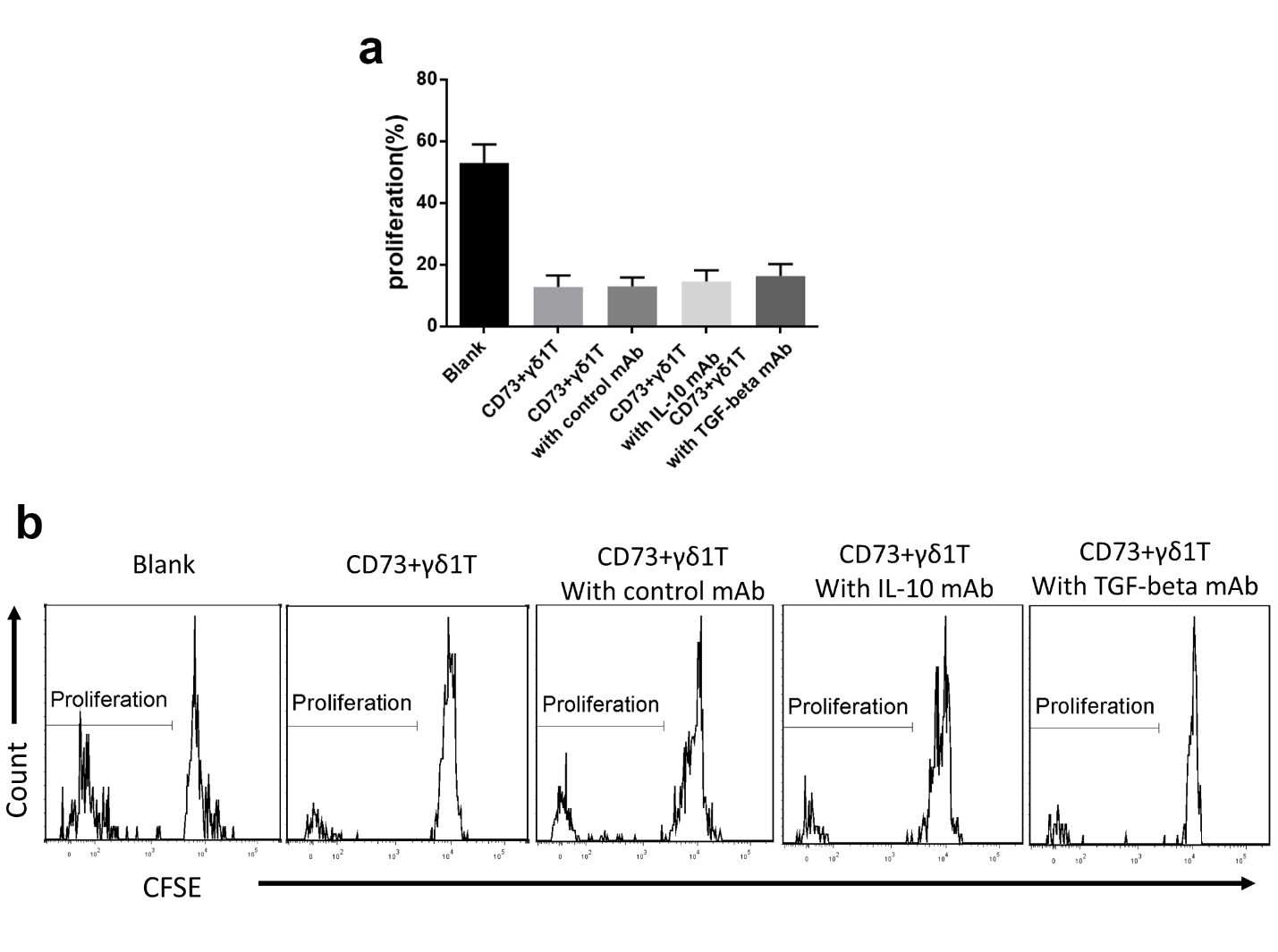
Figure. S5. IL-10 and TGF-β are not involved in the immunosuppressive activity of CD73+γδ Tregs.


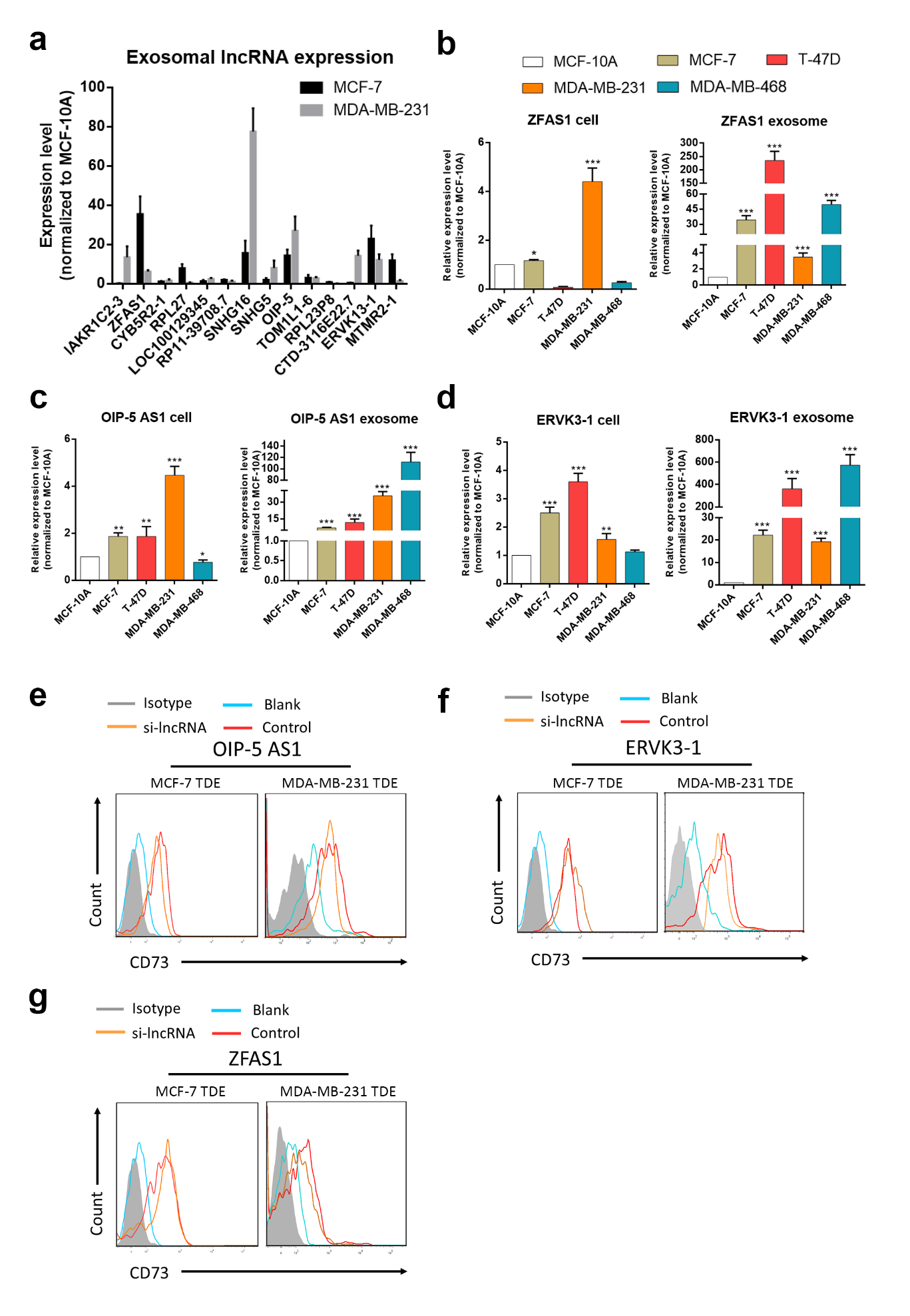
Figure. S6. The effect of candidate lncRNAs on CD73 expression in γδ1 T cells.


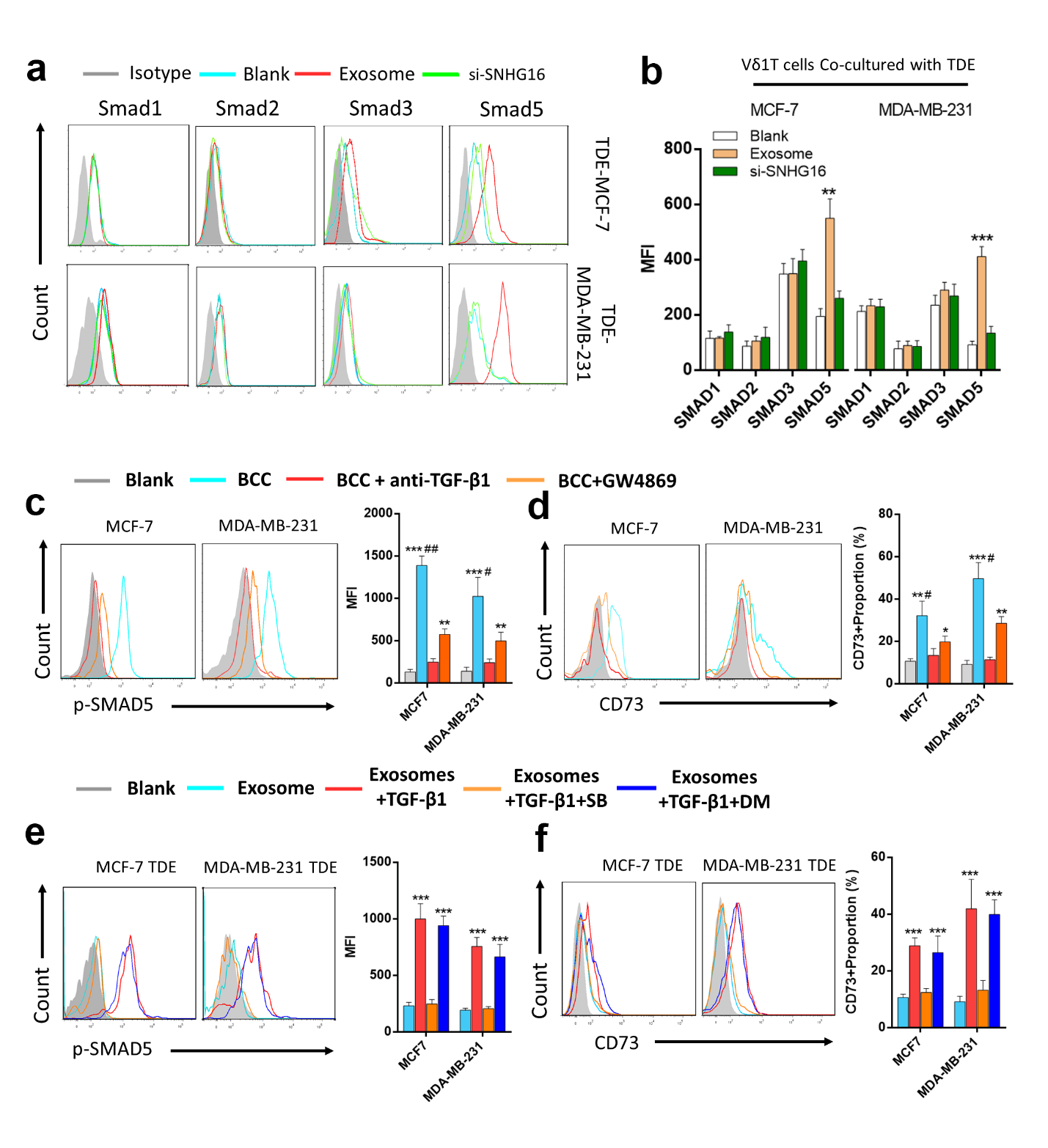
Figure. S7. Breast TDEs induce SMAD5 upregulation in Vδ1 T cells via SNHG16.


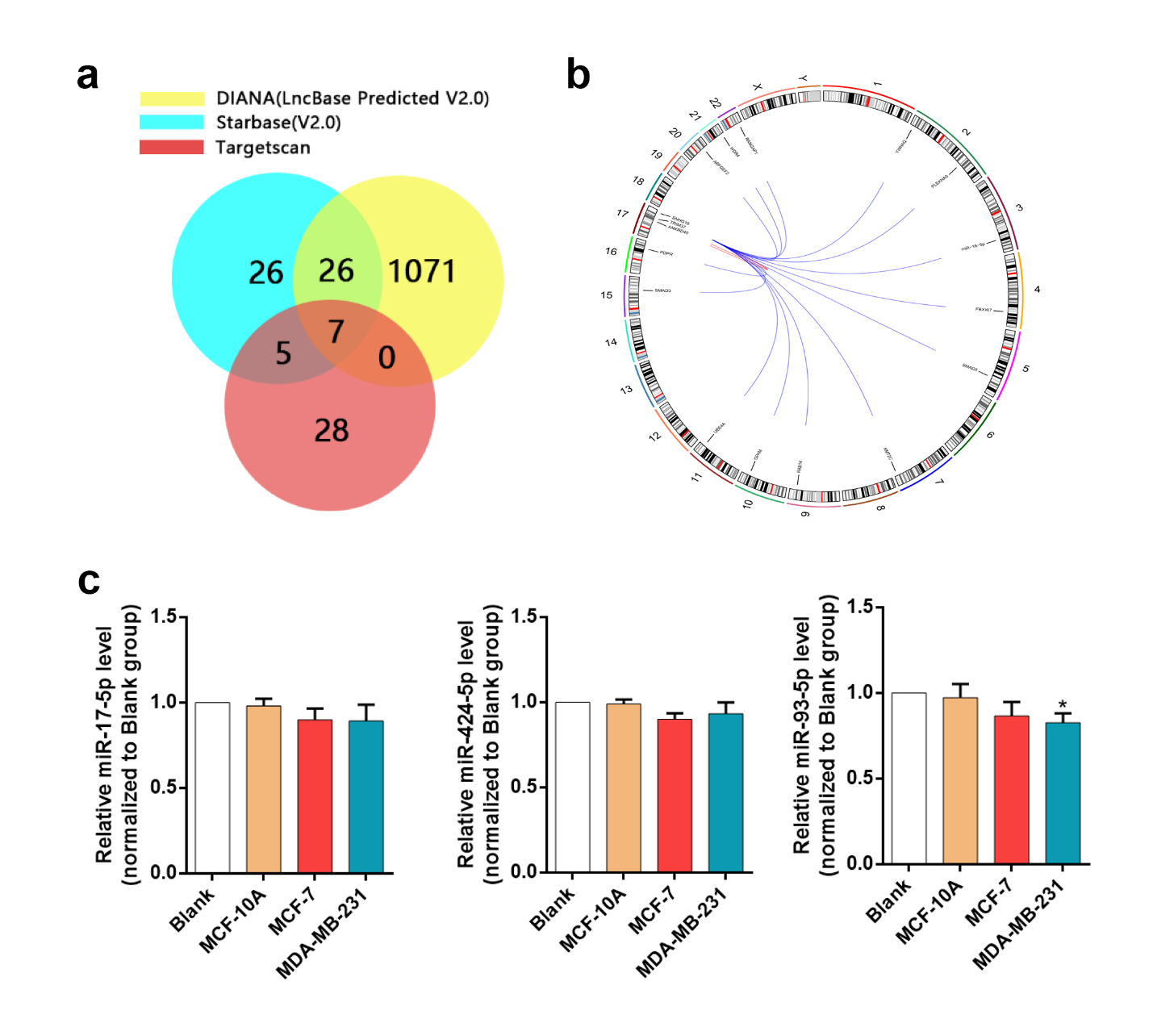
Figure. S8. Prediction of the SNHG16/miRNA/SMAD5 ceRNA regulatory axis.


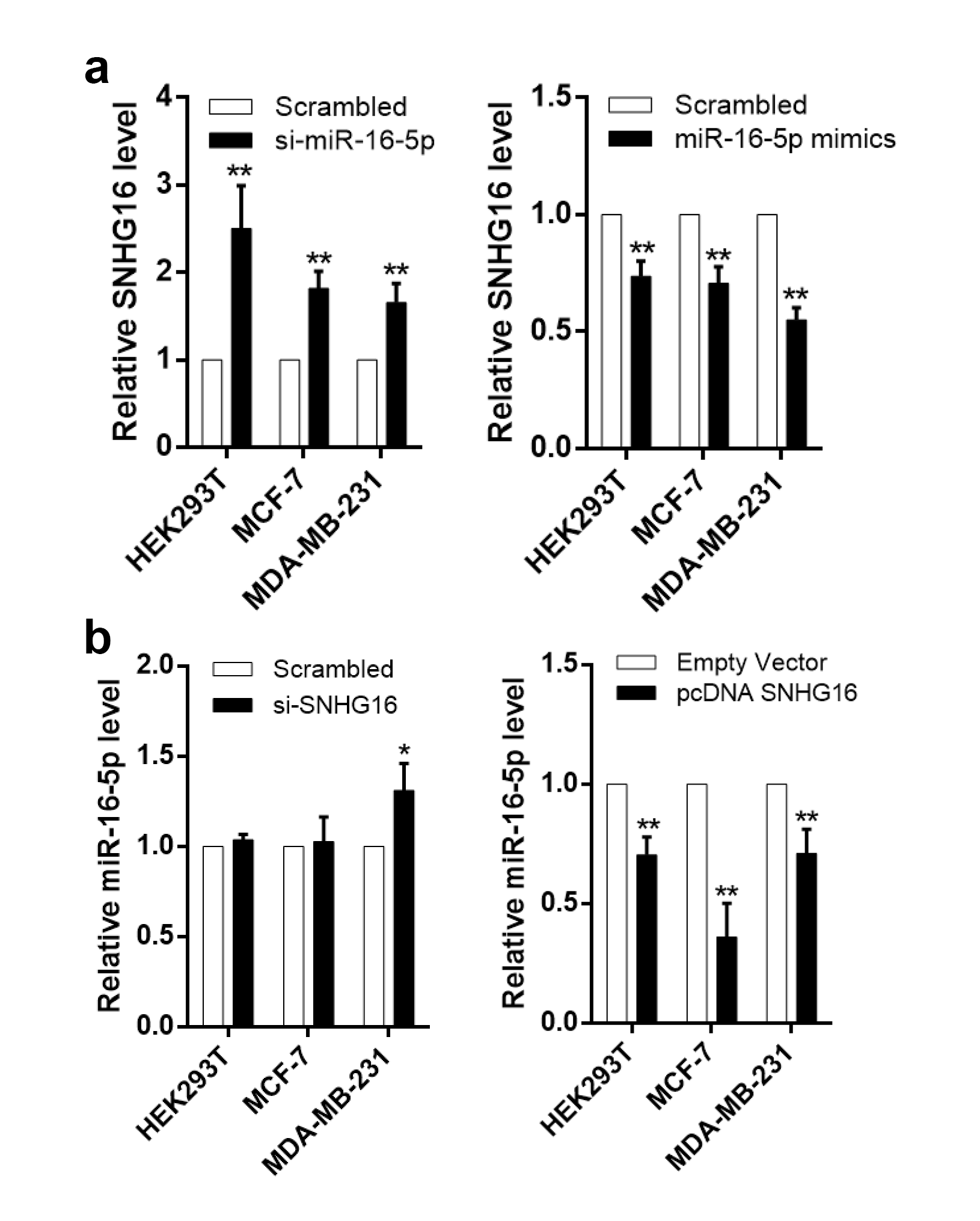
Figure. S9. Regulatory relationship between SNHG16 and miR-16-5p.


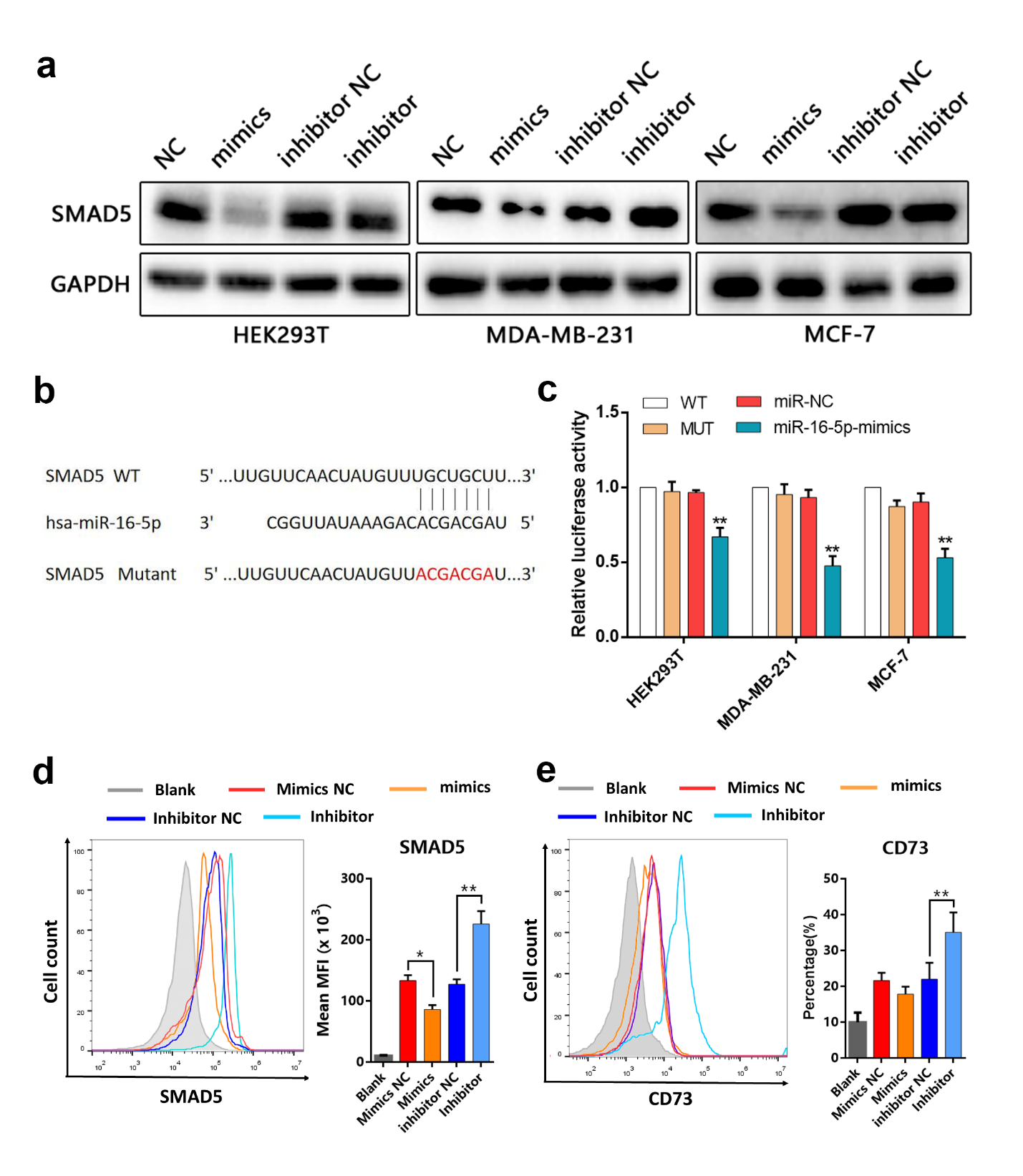
Figure. S10. SMAD5 is a direct target gene of miR-16-5p.

| **Antibodies for flow cytometry** | **Manufacturers** |
| --- | --- |
| Anti-CD3 FITC/APC/PE-CY7 | Biolegend |
| Anti-CD4 FITC/PE | Biolegend |
| Anti-CD8 APC/Percp | Biolegend |
| Anti-CD25 FITC | Biolegend |
| Anti-CD39 FITC/PE-CY7 | Biolegend |
| Anti-CD45 FITC/Percp-Cy5.5 | Biolegend |
| Anti-CD73 APC/APC-Cy7 | Biolegend |
| Anti-Vδ2 Percp | Biolegend |
| Anti-Vδ2 APC | Miltenyi Biotec |
| Anti-Vδ1 FITC | Genetex |
| Anti-IL-4 PE | Biolegend |
| Anti-IL-10 PE | Biolegend |
| Anti-IL-17A Percp | Biolegend |
| Anti-Foxp3 PE | Biolegend |
| Anti-GM-CSF PE | Biolegend |
| Anti-IFN-γ PE-CY7 | Biolegend |
| Anti-PD-L1 APC | Biolegend |
| Anti-CD122 PE | Biolegend |
| Anti-TGF-β1 PE | Biolegend |
| Anti-Perforin APC-cy7 | Biolegend |
| Anti-Granzyme B PE-Cy7 | Biolegend |
| Anti-FasL PE | Biolegend |
| Anti-TRAIL PE | Biolegend |
| Anti-Nkp44 PE-cy7 | Biolegend |
| Anti-Nkp46 Percp-Cy5.5 | Biolegend |
| Anti-Smad2 unconjugated (Clone 31H15L4) | Invitrogen |
| Anti-Smad3 unconjugated (Clone SY25-01) | Invitrogen |
| Anti-Smad4 unconjugated Polyclone | Invitrogen |
| Anti-Smad5 unconjugated Polyclone | ProteinTech |
| Anti-phospho-Smad5 unconjugated (Clone 31H14L11) | Invitrogen |
| FITC/APC Donkey anti-rabbit IgG antibody | Biolegend |

**Table S1.** Antibodies used for flow cytometry.

| Gene name | Forward Primer | Reverse Primer |
| --- | --- | --- |
| CD73 | 5’- TGGAACCACGTATCCATGTG-3’ | 5’- ATGCTCAAAGGCCTTCTTCA-3’ |
| miR-16-5p | 5’- CTCAACTGGTGTCGTGGAGTCGGCAATTCAGTTGAGCGCCAATA- 3’ | 5’- TCGGCAGGTAGCAGCACGTAAA -3’ |
| miR-17-5p | 5′-AGTCCGTATCTTCATTCCGCCTAGC-3 | 5′-AAGCTGCGTAAGGATACTTGCGA-3′ |
| miR-93-5p | 5’-GCCATGTAAACATCTCGGACTG-3’ | 5’-CAATGCGTGTGGTGGAGGAG-3’ |
| miR-424-5p | 5′‐TTTATTCACCCGCAGGTACCCC‐3′ | 5′‐GCAGACCCCACCTTCTACCT‐3′ |
| SNHG16 | 5’-GAAGCGAGCTGAGAGGCTTT-3’ | 5’- GGGGAAACCATGGCATTCTG-3’ |
| ZFAS1 | 5’- CCAGGATGAATGCGGGTGT-3’ | 5’- AGGCAGACTGAATCAAGCCAA-3’ |
| OIP-5 AS1 | 5’- CTGCGAAGATGGCGGAGTAA-3’ | 5’- TTCCTCTCCTCTGGCCGATA-3’ |
| ERVK3-1 | 5’- GGCAGTGTGTAAGACGCTCA-3’ | 5’- CCACTTGTCACGGTTGCTTC-3’ |
| U3 | 5′‐CCACGAGGAAGAGAGGTAGC-3′ | 5′‐CACTCAGACCGCGTTCTCTC-3′ |
| U6 | 5’- CTCGCTTCGGCAGCACA-3’ | 5’- AACGCTTCACGAATTTGCGT-3’ |
| GAPDH | 5’- GATTTGGTCGTATTGGGCG-3’ | 5’- TGGAAGATGGTGATGGGAT-3’ |

Table S2. RT-PCR primers used in this study

| **Name** | **Sequence (5’ to 3’)** |
| --- | --- |
| **mimic** |  |
| miR-16-5p-mimic | UAGCAGCACGUAAAUAUUGGCG |
| miR-17-5p-mimic | CAAAGUGCUUACAGUGCAGGUAG |
| miR-195-5p-mimic | UAGCAGCACAGAAAUAUUGGC |
| miR-497-5p-mimic | CAGCAGCACACUGUGGUUUGU |
| miR-93-5p-mimic | CAAAGUGCUGUUCGUGCAGGUAG |
| miR-106b-5p-mimic | TAAAGTGCTGACAGTGCAGAT |
| miR-424-5p-mimic | CAGCAGCAAUUCAUGUUUUGAA |
| **inhibitor** |  |
| miR-16-5p-inhibitor | CGCCAAUAUUUACGUGCUGCUA |
| Negative control | CAGUACUUUUGUGUAGUACAA |

Table S3. Sequence of miRNA mimic or inhibitor.

Table Clinical factors grouped by quantiles of γδT cell relative abundance

| Clinical factors | | γδT cell relative abundance | | | P value |
| --- | --- | --- | --- | --- | --- |
|  |  | lower quantile | middle quantile | upper quantile |  |
| Age (yr) |  | 49.0±11.1 | 49.0±11.5 | 49.0±12.4 | 0.256 |
| T stage | T1 | 53(30%) | 59(27%) | 63(31%) | 0.1609 |
| (No./%) | T2 | 116(63%) | 133(60%) | 117(57%) | |
|  | T3 | 8(4%) | 23(10%) | 14(7%) | |
|  | T4 | 6(3%) | 5(3%) | 11(5%) | |
| N stage | N0 | 107(49%) | 90(37%) | 93(41%) | 2.20E-16 |
| (No./%) | N1-3 | 111(51%) | 156(63%) | 135(59%) | |

Table Multivariable Cox-regression survival analysis (overall survival, OS; N=405)

| Clinical factors | HR | 95% CI | P value |
| --- | --- | --- | --- |
| Age | 1.000 | 0.98-1.02 | 0.9625 |
| T stage | 1.581 | 1.17-2.14 | 0.0029 |
| N stage | 1.598 | 1.31-1.96 | 5.58E-06 |
| γδ T relative abundance | 152.344 | 1.06-21943.39 | 0.0475 |

Table Multivariable Cox-regression survival analysis (disease-free survival, DFS; N=233)

| Clinical factors | HR | 95% CI | P value |
| --- | --- | --- | --- |
| Age | 1.00 | 0.99-1.02 | 0.5591 |
| T stage | 1.22 | 0.86-1.73 | 0.2694 |
| N stage | 1.27 | 0.77-2.10 | 0.3509 |
| γδ T relative abundance | 736.84 | 8.35-65026.14 | 0.0039 |

Table S4. Clinical factors grouped based on the tertiles of γδT cell relative abundance and multivariable Cox regression survival analysis
